# A *Recql5* mutant facilitates complex CRISPR/Cas9-mediated chromosomal engineering in mouse zygotes

**DOI:** 10.1093/genetics/iyae054

**Published:** 2024-04-05

**Authors:** Satoru Iwata, Miki Nagahara, Risako Ido, Takashi Iwamoto

**Affiliations:** Center for Education in Laboratory Animal Research, Chubu University, Kasugai, Aichi 487-8501, Japan; Department of Biomedical Sciences, College of Life and Health Sciences, Chubu University, Kasugai, Aichi 487-8501, Japan; College of Bioscience and Biotechnology, Chubu University, Kasugai, Aichi 487-8501, Japan; Center for Mathematical Science and Artificial Intelligence, Chubu University, Kasugai, Aichi 487-8501, Japan; Center for Education in Laboratory Animal Research, Chubu University, Kasugai, Aichi 487-8501, Japan; Department of Biomedical Sciences, College of Life and Health Sciences, Chubu University, Kasugai, Aichi 487-8501, Japan; Center for Education in Laboratory Animal Research, Chubu University, Kasugai, Aichi 487-8501, Japan; Department of Biomedical Sciences, College of Life and Health Sciences, Chubu University, Kasugai, Aichi 487-8501, Japan

**Keywords:** chromoanasynthesis, complex chromosomal rearrangements, CRISPR/Cas9, *Recql5*

## Abstract

Complex chromosomal rearrangements (CCRs) are often observed in clinical samples from patients with cancer and congenital diseases but are difficult to induce experimentally. Here, we report the first success in establishing animal models for CCRs. Mutation in *Recql5*, a crucial member of the DNA helicase RecQ family involved in DNA replication, transcription, and repair, enabled CRISPR/Cas9-mediated CCRs, establishing a mouse model containing triple fusion genes and megabase-sized inversions. Some of these structural features of individual chromosomal rearrangements use template switching and microhomology-mediated break-induced replication mechanisms and are reminiscent of the newly described phenomenon “chromoanasynthesis.” These data show that *Recql5* mutant mice could be a powerful tool to analyze the pathogenesis of CCRs (particularly chromoanasynthesis) whose underlying mechanisms are poorly understood. The *Recql5* mutants generated in this study are to be deposited at key animal research facilities, thereby making them accessible for future research on CCRs.

## Teaser

Researchers have created animal models to study complex chromosomal rearrangements linked to cancer and congenital diseases by altering the *Recql5* gene in mice. This breakthrough, using CRISPR/Cas9 technology, allows for detailed examination of genetic disruptions often seen in these conditions. The genetically modified mice offer a new resource for understanding and researching complex genetic issues.

## Introduction

Recent advances in bioinformatics technologies have led to the detection of complex chromosomal rearrangements (CCRs) consisting of ≥3 chromosomal breaks in patients with cancer and congenital diseases (Pellestor 2019). These rearrangements caused by catastrophic cellular events can affect the phenotype, thereby inducing a disease-promoting environment (Burssed et al. 2022). In particular, chromoanasynthesis is a recently discovered form of CCRs. It involves complex rearrangements that are caused by erroneous DNA replication of a single chromosome through fork stalling and template switching (FoSTeS) and microhomology-mediated break-induced replication (MMBIR; Pellestor and Gatinois 2018). However, the pathogenic mechanisms underlying the diseases caused by CCRs remain unclarified, and the establishment of appropriate animal models is essential for their elucidation. Although recent technologies, such as clustered regularly interspaced short palindromic repeats (CRISPR)–dependent base editing, prime editing, and DNA integration, have allowed for high-precision genome interrogation (Nambiar et al. 2022), they were not adapted to model CCRs in the germ line.

Here, we hypothesized that the efficient induction of CCRs could be achieved by manipulating the DNA repair pathway because accumulating evidence indicates that changes in DNA repair timing often accompany genomic rearrangements (Burssed et al. 2022). However, these strategies may have adverse consequences given the importance of DNA repair genes in genome maintenance. Notably, most genes involved in the DNA repair pathway are essential, and their homozygous disruption leads to embryonic lethality in mice (Menolfi and Zha 2020). However, *Recql5-*deficient mice live to adulthood (Hu et al. 2007). RecQ protein-like 5 (RECQL5) helicases can displace the DNA repair protein RAD51 from single-stranded (ss) DNA and disassemble nucleoprotein filaments, thereby suppressing homology-directed repair (HDR; Hu et al. 2007). Transient accumulation of the homologous recombination (HR) and RAD51 has been reported in *Recql5*-deficient cells (Hu et al. 2007; Paliwal et al. 2014), which could alter DNA repair pathways, thereby contributing to chromosomal rearrangements. RECQL5 also interacts with RNA polymerase II (RNAPII), negatively regulating transcript elongation (Kanagaraj et al. 2010). Recent insights also suggest the involvement of RECQL5 in RNA polymerase I (RNAPI), indicating its importance in rRNA gene stability and transcription, which is essential for ribosomal biogenesis (Urban et al. 2016). This interaction suggests a broad role of RECQL5 in genomic stability, not only by modulating DNA repair mechanisms but also by influencing the transcription processes. Additionally, studies on *Recql5*-deficient mice have demonstrated increased levels of sister chromatid exchange and a heightened susceptibility to various cancers, underscoring the critical role of RECQL5 in genome stability maintenance (Hu et al. 2005). Given these multifaceted roles of RECQL5, we investigated the potential induction of CCRs in *Recql5* mutant mice and successfully established a CCR model. Notably, a DNA repair system (FoSTeS/MMBIR) was involved in the CCR model mouse line.

## Materials and methods

### Experimental animals

Wild-type (WT; C57BL/6NCrSlc) mice (Japan SLC, Shizuoka, Japan) were used in this study. The animals were housed at a constant temperature (22 ± 2°C) and humidity (50 ± 10%), with a 12-h light/12-h dark cycle. All animal experiments were approved by the Institutional Animal Care and Use Committee of Chubu University (Permit Number #202110033) and were conducted in accordance with the institutional guidelines.

### CRISPR RNP and single-stranded oligodeoxynucleotide preparation

The CRISPR guide RNAs were designed using CHOPCHOP (Labun et al. 2019; http://chopchop.cbu.uib.no/; Supplementary Table 6). CRISPR RNP consists of Alt-R S.p. Cas9 Nuclease 3NLS (Integrated DNA Technologies, Coralville, IA, USA) and a custom guide RNA (crRNA): universal structural RNA (tracrRNA) duplex (Integrated DNA Technologies). The crRNA and tracrRNA were resuspended in Nuclease-Free Duplex Buffer (Integrated DNA Technologies, Coralville, IA, USA) to achieve a final concentration of 4,000 ng/μL. The crRNA and tracrRNA were mixed in equimolar ratios, heated to 95°C for 10 min, and then slowly cooled to 25°C. This crRNA:tracrRNA duplex and the Alt-R S.p. Cas9 Nuclease 3NLS were incubated at 25°C for 10 min to form the RNP complex. The single-stranded oligodeoxynucleotides (ssODNs) were manufactured by Eurofins Genomics (Tokyo, Japan) and designed to join 2 DNA sequences so that the junction would be positioned at the center of the predicted cleavage sites, which were located within 3 bp of the PAM sequences (Supplementary Table 7). The 5′ and 3′ ends of the ssODNs were protected with 2 consecutive phosphorothioate-modified bases (*) to improve the HDR efficiency (Renaud et al. 2016; Supplementary Table 7).

### Improved genome editing via oviductal nucleic acid delivery method

Female mice (8–12-week-old) in estrus were mated with males (8–24-week-old) at 16:00–18:00 h. The presence of copulation plugs was confirmed the next morning via visual inspection, and plug-positive mice were subjected to improved genome editing via oviductal nucleic acid delivery (*i*-GONAD) experiments, as previously described (Ohtsuka et al. 2018; Gurumurthy et al. 2019). To generate chromosomes with an inversion, the following CRISPR solutions were used: 540 ng/μL Alt-R S.p. Cas9 Nuclease 3NLS, 33 μM crRNA:tracrRNA duplex for each of the left and right targets, 150 ng/μL ssODN for each of the left and right targets, and 0.05% Fast Green FCF (Wako, Osaka, Japan) marker diluted in Opti-MEM (Thermo Fisher Scientific, Waltham, MA, USA). To generate CCRs, the following CRISPR solutions were used: 600 ng/μL Alt-R S.p. Cas9 Nuclease 3NLS, 25 μM crRNA:tracrRNA duplex for each of the left, middle, and right targets, 290 ng/μL ssODN for each of the left, middle, and right targets, and 0.05% Fast Green FCF (Wako) marker diluted in Opti-MEM (Thermo Fisher Scientific). Prior to electroporation, females were anesthetized with a mixture of medetomidine (0.75 mg/kg), midazolam (4 mg/kg), and butorphanol (5 mg/kg). The CRISPR mixture (1 μL) was injected into the oviductal lumen upstream of the ampulla using a glass micropipette including a vertical capillary puller (NARISHIGE, Tokyo, Japan). The CRISPR mixture was injected using a FemtoJet 4i microinjector (Eppendorf, Hamburg, Germany) with the following settings: pi: 100 hPa, ti: 0.2 s, and pc: 0 hPa. The oviduct close to the ovary side was clamped with a hemostatic clip (Natsume Seisakusho, Tokyo, Japan) to prevent the CRISPR reagents from flowing back toward the ovary. After injection of CRISPR solutions, the oviduct regions were clamped using tweezer electrodes (LF650P3; BEX, Tokyo, Japan), and electroporation was performed as previously described (Ohtsuka et al 2018; Gurumurthy et al. 2019) using a CUY21EDIT II (BEX, Tokyo, Japan). The following parameters were used for electroporation: square (mA), (+/−), Pd V: 60 or 80 V, Pd A: 200 mA, Pd on: 5.00 ms, Pd off: 50 ms, Pd N: 3, decay: 10%, DecayType: Log. Thereafter, we placed the oviducts back in their original location and sutured the incisions. Post operation, atipamezole hydrochloride (0.75 mg/kg) was intraperitoneally injected to reverse the effects of medetomidine.

### In vitro electroporation

Female mice were intraperitoneally injected with 7.5 IU PMSG (ASKA Animal Health, Tokyo, Japan), followed by 7.5 IU of hCG (ASKA Animal Health, Tokyo, Japan) 48 h later. Thirteen hours after hCG injection, superovulated female mice were euthanized via cervical dislocation, and unfertilized oocytes isolated from the female mice were subjected to in vitro fertilization with freshly isolated spermatozoa from euthanized male mice. To generate chromosomes with an inversion, the following concentrations of CRISPR reagents were used: 100 ng/μL Alt-R S.p. Cas9 Nuclease 3NLS, 6 μM crRNA:tracrRNA duplex for each of the left and right targets, and 100 ng/μL ssODN for each of the left and right targets, all diluted in Opti-MEM (Thermo Fisher Scientific). To generate CCRs, the following concentrations of CRISPR reagents were used: 150 ng/μL Alt-R S.p. Cas9 Nuclease 3NLS, 6 μM crRNA:tracrRNA duplex for each of the left, middle, and right targets, and 100 ng/μL ssODN for each of the left, middle, and right targets, all diluted in Opti-MEM (Thermo Fisher Scientific). The in vitro electroporation procedures were performed as previously described (Kaneko et al. 2014). Briefly, the embryos were cultured in KSOM (Kyudo, Saga, Japan), washed with Opti-MEM, and then placed in an electrode cuvette (CUY505P5, NEPA GENE, Chiba, Japan) with CRISPR solutions (total volume, 47 μL), followed by electroporation using a NEPA21 (NEPA GENE). The following parameters were used for electroporation: poring pulse (voltage: 225 V; pulse length: 2.0 ms; pulse interval: 50 ms; number of pulses: 4; decay rate: 40%; polarity: +) and transfer pulse (voltage: 20 V; pulse length: 50 ms; pulse interval: 50 ms; number of pulses: 5; decay rate: 40%; polarity: ±). After electroporation, the embryos were cultured to the blastocyst stage in KSOM.

### Analysis of CRISPR/Cas9-engineered mice

To screen for CRISPR/Cas9-induced mutations, genomic DNA was isolated from the tails or ears of the founder mice using the Kaneka Easy DNA Extraction Kit version 2 (Kaneka, Tokyo, Japan). The DNA was examined by PCR amplification, utilizing the EmeraldAmp PCR Master Mix (Takara Bio, Shiga, Japan) under the following conditions: 30 cycles of denaturation at 98°C for 10 s, annealing at 60°C for 30 s, and extension times varying according to the primer used—either 30 s, 1 min, or 1 min and 30 s—with a final indefinite hold at 10°C. The obtained PCR products were purified using a NucleoSpin Gel and PCR Cleanup kit (Takara Bio, Shiga, Japan) and sequenced directly or cloned into the pTAC-1 vector (Biodynamics, Tokyo, Japan). The sequences of individual clones were determined using Sanger sequencing (Eurofins Genomics). The PCR primers used for genotyping are listed in Supplementary Table 8.

### DNA extraction from mouse blastocysts

Crude DNA derived from each blastocyst was extracted using the Kaneka Easy DNA Extraction Kit version 2 (Kaneka, Tokyo, Japan). Briefly, we collected blastocysts in a Petri dish and transferred a single blastocyst to a 0.2-mL PCR tube using a glass micropipette. Subsequently, 10 μL of solution A was directly added to each tube, and the samples were incubated at 98 °C for 8 min. After cooling, 1.4 μL of solution B was added to each tube, followed by thorough mixing by pipetting. Whole-genome amplification was performed using the commercially available illustra GenomiPhi V2 DNA Amplification Kit (GE Healthcare Life Sciences, Piscataway, NJ, USA), according to the manufacturer's instructions to increase the total genomic DNA amount. The DNA was subsequently examined by PCR amplification, utilizing the EmeraldAmp PCR Master Mix (Takara Bio, Shiga, Japan). For nested PCR, the first round consisted of 40 cycles of denaturation at 98°C for 10 s, annealing at 63°C for 30 s, and extension at 72°C for 1 min, followed by a final indefinite hold at 10°C. The second round of PCR involved 15 cycles of denaturation at 98°C for 10 s, annealing at 63°C for 30 s, and extension at 72°C for 30 s, with a final indefinite hold at 10°C. The obtained PCR products were purified using the NucleoSpin Gel and PCR Cleanup kit (Takara Bio, Shiga, Japan) and cloned into the pTAC-1 vector (Biodynamics, Tokyo, Japan). The sequences of individual clones were determined using Sanger sequencing (Eurofins Genomics). The PCR primers used for genotyping are listed in Supplementary Table 8.

### RT-PCR

RT-PCR was performed using total RNA. Total RNA was isolated from ear tissue using ISOSPIN Cell & Tissue RNA (Nippon Gene, Tokyo, Japan). Template cDNA was obtained using ReverTra Ace qPCR RT Master Mix (Toyobo, Osaka, Japan). The RT-PCR products were purified using a NucleoSpin Gel and PCR Cleanup kit (Takara Bio) and directly analyzed using Sanger sequencing (Eurofins Genomics). The primers used for RT-PCR are listed in Supplementary Table 8.

### Quantitative RT-PCR

Total RNA was isolated from ovary and testis tissue using ISOSPIN Cell & Tissue RNA (Nippon Gene, Tokyo, Japan). Standard quantitative RT-PCR was performed with SYBR Green as the dye. Briefly, 1 μg of total RNA was used for reverse transcription with ReverTra Ace qPCR RT Master Mix (Toyobo, Osaka, Japan), after which quantitative PCR was performed using KOD SYBR qPCR Mix (Toyobo, Osaka, Japan) on a CFX96 Touch Real-Time PCR Detection System (Bio-Rad Laboratories, CA, USA). All values were corrected by each calibration curve, and the relative expression level was measured with the ΔΔCt method using the *Ywhaz* gene for normalization. All samples were analyzed in triplicates. Primers for quantitative RT-PCR are listed in Supplementary Table 8.

### Western blot analysis

Western blot analysis was performed as described previously (Ogawa et al. 2020). The membrane was analyzed with FUSION-SOLO 4S.WL (Vilber Lourmat, France), and GAPDH was used for normalization. The antibodies used in this study were as follows: anti-RECQL5 (#sc-515050, 1:500 dilution; Santa Cruz Biotechnology, TX, USA), anti-GAPDH (#60004, 1:2,000 dilution, Proteintech, IL, USA), and anti-mouse IgG HRP-linked antibody (#7076P2, 1:1,000 dilution; Cell Signaling Technology, MA, USA).

### AlphaFold2 analysis

The predicted 3D structure of the *Recql5^em1/em1^* mutant was analyzed compared to the WT protein structure. The prediction of both protein structures was performed using AlphaFold2 (Jumper et al. 2021; https://colab.research.google.com/github/sokrypton/ColabFold/blob/main/AlphaFold2.ipynb, accessed on 2023 June 20). The structural figures were generated using PyMoL (https://pymol.org, accessed on 2023 April 12).

### Growth analysis

The weights (g) of *CCRs(10)^#4a/#4a^*, *CCRs(10)^#4a/+^*, and WT (+/+) mice were recorded for growth curve analysis from 0 to 12 weeks after birth.

### Food intake analysis

Mice were housed individually, and food intake was measured in terms of grams of diet consumed per day.

### Mating test

Upon sexual maturation, male mice were caged with 2 females for at least 8 weeks. During the mating test, pups were counted for litter size measurements, and their tails or ears were biopsied for genotyping.

### Whole-genome sequencing analysis

Total genomic DNA was extracted using a DNeasy Blood & Tissue Kit (Qiagen, Hilden, Germany). The library was prepared using TruSeq Nano DNA (Illumina, CA, USA) according to the manufacturer's library quantification protocol. Sequencing was performed as pair-end (150 bp) using an Illumina NovaSeq6000 (Illumina) by the Macrogen sequencing service (Macrogen Inc., Seoul, Korea). The whole-genome sequencing (WGS) analysis and genomic coordinates provided in this manuscript are based on the GRCm38/mm10 assembly. The sequencing data were aligned in BAM format to the mouse reference genome (mm10) using the Integrative Genomics Viewer (IGV, version 2.3.93; Thorvaldsdóttir et al. 2013). Structural rearrangements and breakpoints were identified using Manta Structural Variant Caller (Chen et al. 2016b; version 1.3.1). Breakpoints were visually inspected using IGV (version 2.3.93) to confirm the presence of split and spanning reads.

### Quantification and statistical analysis

Student's *t*-test (2-tailed test) was used for quantitative RT-PCR, body weight, food intake, and mating analyses. Differences in Mendelian genotype ratios of progeny obtained from sibling mating between CCRs mice were tested using the chi square test. Statistical comparisons were made using Tukey's honestly significant difference test. Statistical analysis of chromosomal rearrangement frequencies and blastocyst development rates were performed using the Mann–Whitney *U* test (2-tailed test). Data are presented as the mean ± standard deviation. The threshold for statistical significance was *P* < 0.05. All statistical analyses were performed using Excel version 16.36 (Microsoft, Redmond, WA, USA) and MATLAB R2023b (MathWorks, Natick, MA, USA).

## Results and discussion

### *Recql5* mutant enables CRISPR/Cas9-mediated CCRs in mouse zygotes

Mutations within the RAD51-binding domain abolish the physical interaction between RECQL5 and RAD51, significantly impairing the ability of RECQL5 to disrupt RAD51-ssDNA filaments in vitro and hindering HR-mediated accurate DSB repair in vivo (Schwendener et al. 2010; Islam et al. 2012). This disruption in RAD51 interaction leads to the transient accumulation of RAD51 (Hu et al. 2007; Paliwal et al. 2014), potentially altering DNA repair pathways and contributing to chromosomal rearrangements. A previously developed in vivo electroporation technique, called *i*-GONAD (Ohtsuka et al. 2018; Gurumurthy et al. 2019; Supplementary Fig. 1), was used to establish a mouse strain with a deletion of the RAD51-binding domain in RECQL5 (*Recql5^em1^*; Supplementary Fig. 2a and b). This approach resulted in a frameshift mutation, culminating in the introduction of a premature stop codon. Notably, *Recql5^em1^* lacks not only the RAD51-binding and Set2–Rpb1-interacting (SRI) domains but also a part of the internal RNAPII-interacting (IRI) domain. The loss of the IRI and SRI domains in RECQL5, crucial for binding with RNA polymerases, disrupts the resolution of conflicts between replication and transcription, potentially leading to increased genomic instability (Urban et al. 2016). Quantitative RT-PCR analysis revealed that RNA expression levels of the *Recql5* gene were significantly lower in *Recql5^em1/em1^* than in the WT (Supplementary Fig. 2c). Consistent with this analysis, we barely detected the native RECQL5 band (the predicted size is 108 kDa) or the mutated band (the predicted size is 58 kDa) in western blotting analysis of the *Recql5^em1/em1^* mutants (Supplementary Fig. 2d). We detected the structural consequences of this deletion using AlphaFold2 analysis (Jumper et al. 2021), which demonstrated the absence of domains downstream of the IRI domain in *Recql5^em1/em1^* (Supplementary Fig. 2e). These findings suggest that *Recql5^em1/em1^* mice may represent a knockdown line in which either an increase in nonsense-mediated decay at the mRNA level, protein instability, or both was induced. These *Recql5^em1/em1^* mice were fertile and unexpectedly showed no overt signs of other diseases, such as tumorigenesis or inflammation, which have been previously reported (Hu et al. 2007; Supplementary Fig. 2f and g); 5 male and 4 female mice were observed for over 100 weeks.

**Fig. 1.**
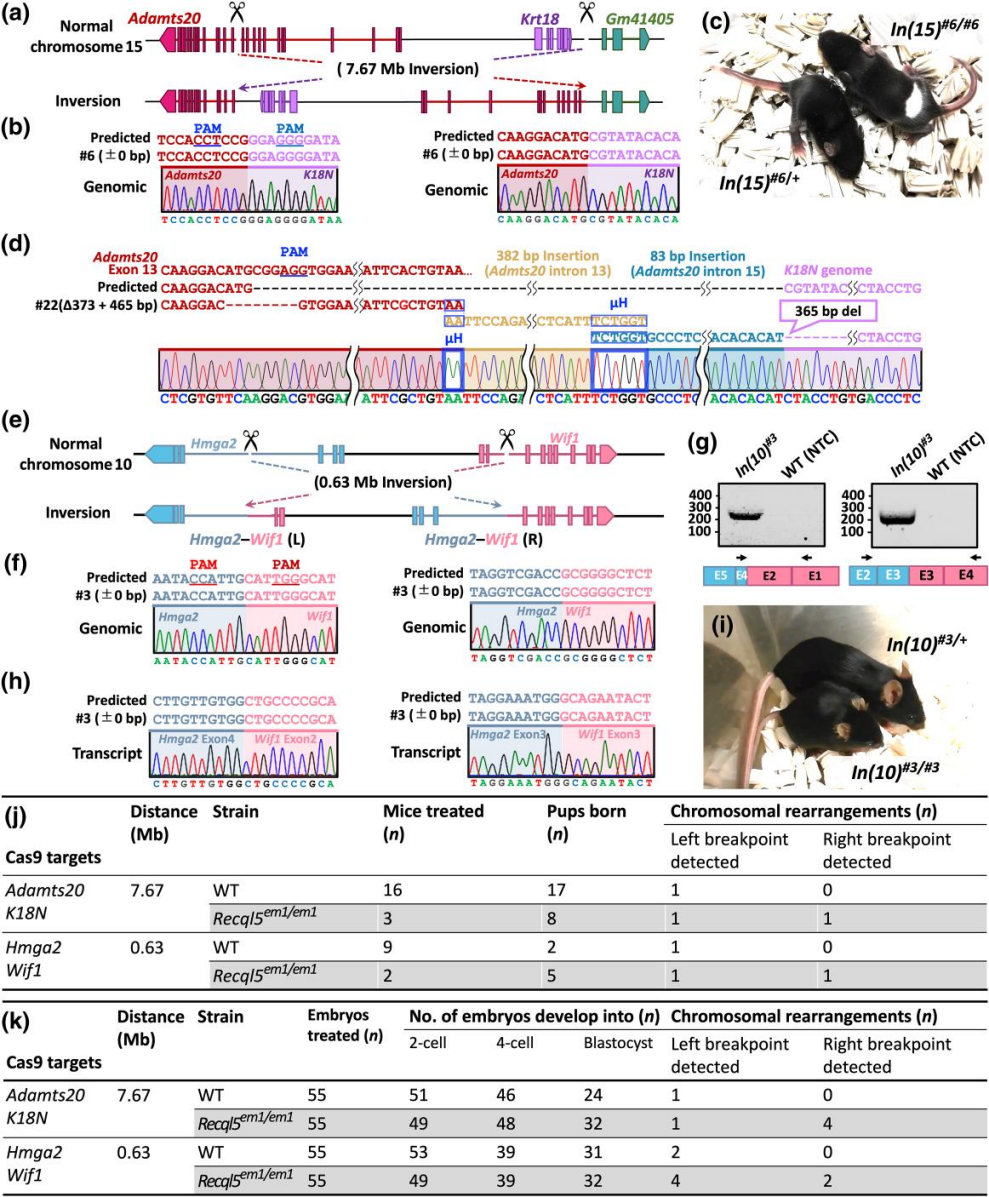
Chromosomal engineering of inversions using the *Recql5* mutant. a) Schematic illustration of the chromosomal rearrangements created by an inversion between *Adamts20* and the K18N-locus in chromosome 15. b) Alignment of sequences corresponding to the *Adamts20* and the K18N-locus genomic breakpoint junctions, obtained via the *i*-GONAD technique. c) The generated inversion [*In(15)^#6^*] on chromosome 15 shows a recessive, white-spotted phenotype. d) Junction sequence obtained through in vitro electroporation, demonstrating complex rearrangements with insertions associated with the FoSTeS/MMBIR mechanism. e) Schematic illustration of the chromosomal rearrangements created by an inversion between the *Hmga2* and *Wif1* genes in chromosome 10. f) Alignment of sequences corresponding to *Hmga2* and *Wif1* genomic breakpoint junctions, obtained via the *i*-GONAD technique. g) PCR amplification of the predicted fusion transcript. NTC, negative control. h) Sanger sequences corresponding to *Hmga2–Wif1* cDNA. i) The generated inversion [*In(10)^#3^*] on chromosome 10 showed a recessive pygmy phenotype. j) Summary of the experimental efficiency of chromosomal inversions using the *i*-GONAD technique. k) Summary of chromosomal inversion efficiency and embryonic development stages post in vitro electroporation.

Second, we investigated the efficacy of utilizing the *Recql5* mutant strategy in the generation of inversion rearrangement mouse models via the *i*-GONAD technique. We recently induced a 7.67-Mb inversion in WT mice (Iwata et al. 2019). We induced this large inversion in *Recql5^em1/em1^* mice with higher genome editing efficiency than that in WT mice (Fig. 1a, b, and j; Supplementary Table 1). Homozygous inversion mice [*In(15)^#6^*] were generated by breeding heterozygous males and females (Fig. 1c). These mice exhibited a white-spotted phenotype owing to disrupted *Adamts20* expression. Subsequently, we employed in vitro electroporation targeting the same genomic regions in isolated zygotes from WT and *Recql5^em1/em1^* strains to further validate our findings. In vitro electroporation was performed as described in a previous study (Kaneko et al. 2014). This approach allowed for an accurate evaluation of chromosomal rearrangements at the individual zygote level. The *Recql5* mutant zygotes exhibited a higher frequency of chromosomal rearrangements at the right breakpoints than the WT counterparts (Fig. 1k; Supplementary Table 1). Sequence determination of the breakpoints revealed the presence of multiple microhomology patterns and alternative templates (Fig. 1d). These findings suggest the involvement of processes used to switch to nearby templates in response to the stalling and collapse during DNA repair, known as FoSTeS and MMBIR (Lee et al. 2007; Holland and Cleveland 2012; Ottaviani et al. 2014). This structural feature of individual chromosomes is reminiscent of the newly described phenomenon chromoanasynthesis, which has been observed in tumors as well as in patients with congenital diseases (Pellestor and Gatinois 2018).

Furthermore, we efficiently modeled the *Hmga2–Wif1* inversion on chromosome 10 in *Recql5^em1/em1^* mice using the *i*-GONAD technique (Fig. 1e, f, and j;  Supplementary Table 2). The human *HMGA2–WIF1* fusion gene that was generated by inversion on chromosome 12 activates the Wnt/β-catenin pathway and is found in salivary gland tumors and breast adenomyoepitheliomas (Pareja et al. 2019). The resultant mice [*In(10)^#3^*] invariably exhibited the *Hmga2–Wif1* inversion. These mice expressed the *Hmga2–Wif1* fusion gene (Fig. 1g and h) and exhibited a recessive pygmy phenotype as a consequence of the *Hmga2* mutation (Lee et al. 2022; Fig. 1i). Following in vitro electroporation, *Recql5* mutant zygotes exhibited a higher frequency of chromosomal rearrangements than the WT counterparts (Fig. 1k; Supplementary Table 2). This finding confirmed the enhanced genome editing capabilities of the *Recql5* mutant approach. Taken together, these results indicate that the *Recql5* mutant approach can efficiently generate inversion mouse models.

**Fig. 2.**
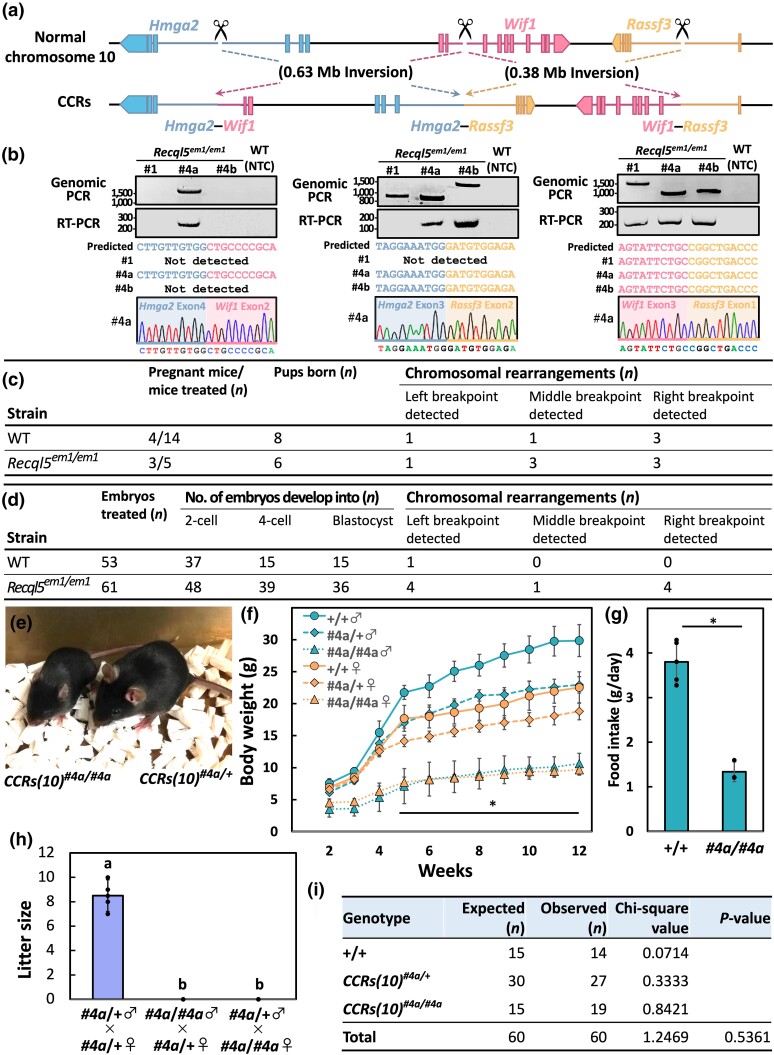
Induction of *Hmga2*–*Wif1*, *Hmga2*–*Rassf3*, and *Wif1*–*Rassf3* complex rearrangements in mouse zygotes using the *Recql5* mutant. a) Schematic representation of the CCRs created between *Hmga2*, *Wif1*, and *Rassf3* in chromosome 10. b) PCR amplification of the predicted breakpoint junction and fusion transcript. Sanger sequences corresponding to *Hmga2–Wif1*, *Hmga2–Rassf3*, and *Wif1–Rassf3* cDNA. NTC, negative control. c) Summary of the experimental efficiency of CCRs using the *i*-GONAD technique. d) Summary of CCR efficiency and embryonic development stages post in vitro electroporation. e) Appearance of heterozygous and homozygous *CCRs(10)^#4a^* male mice at 6 weeks of age. f) Growth curves of WT (+/+) (*n* = 11/10), heterozygous *CCRs(10)^#4a^* (#4a/+) (*n* = 7/8), and homozygous *CCRs(10)^#4a^* (#4a/#4a) (*n* = 9/12) mice (female/male). Error bars, mean ± SD. *P < 0.05 (2-tailed Student's *t*-test). g) Food intake (g/day) of WT (+/+) and *CCRs(10)^#4a/#4a^* male mice (20 weeks of age). Error bars, mean ± SD. *P < 0.05 (2-tailed Student's *t*-test; *n* = 3∼5). h) Comparison of litter sizes. Error bars, mean ± SD. Different letters above the bars indicate significant differences at *P* < 0.05 by Tukey's honest significance test (HSD; *n* = 6). i) Mendelian ratios of newborn mice from *CCRs(10)^#4a^* heterozygous crossings.

Subsequently, we attempted to apply this technique to produce CCR model mice. We selected an approximately 1.1-Mb region of mouse chromosome 10 containing *Hmga2*, *Wif1*, and *Rassf3* (Fig. 2a) since these 3 genes are involved in human cancer and are mapped in a similar configuration on human chromosome 12. *RASSF3* is an important gene in p53-dependent apoptosis and functions as a tumor suppressor (Kudo et al. 2012). We designed gRNAs targeting these 3 genes and ssODNs that joined the chromosomal breakpoints, each of which had a sequence homologous to each junction point. Thus, 2 inversions were induced by the HDR process between the targeted regions and the homologous ssODNs. The 5′ and 3′ ends of the ssODNs were protected with 2 consecutive phosphorothioate-modified bases to improve the efficiency of HDR (Renaud et al. 2016). We subsequently injected CRISPR/Cas9 ribonucleoproteins (RNPs) targeting these genes into pregnant females to generate chromosomal rearrangements (Ohtsuka et al. 2018; Gurumurthy et al. 2019; Supplementary Fig. 1). The generation of predicted chromosomal rearrangements was initially confirmed by PCR of the genomic DNA and validated by sequencing the corresponding fusion transcript. Here, founder (F0) mice (wherein a central breakpoint was detected) were defined as having induced CCRs and were used for subsequent analyses.

In the *Recql5^em1/em1^* strain, we obtained 6 F0 pups via cesarean section and found that 4 had chromosomal rearrangements in the target locus, yielding 3 viable F0 CCR mice (Fig. 2b and c: #1, #4a, and #4b). In contrast, control WT strains showed partial chromosomal rearrangements in 3 of the 8 pups; however, we could not obtain the surviving founder, F0 (Fig. 2c;  Supplementary Table 3). In subsequent experiments, in vitro electroporation was performed on the *Recql5^em1/em1^* strain, demonstrating enhanced chromosomal rearrangement efficiency compared to the control WT strains (Fig. 2d; Supplementary Table 3). Notably, the proportion of embryos developing into blastocysts in the *Recql5^em1/em1^* strain was higher than that for the WT strains (Fig. 2d). Imprecise repair of DSBs has the potential to be highly deleterious, owing to genomic instability, including the formation of chromosomal rearrangements (Brunet and Jasin 2018). Despite this seemingly difficult chromosomal rearrangement pattern, we confirmed the expression of the corresponding fusion transcript using RT-PCR and direct sequencing (Fig. 2b). Homozygous *CCRs(10)^#4a^* mice, wherein all 3 fusion genes resulted in a recessive pygmy phenotype (including severe growth retardation and infertility) owing to the *Hmga2* mutation (Lee et al. 2022; Fig. 2e–h), did not develop tumors within the timeframe of analysis. These findings suggest that expression of the HMGA2–WIF1 fusion protein does not necessarily play a tumor-promoting role in mice, although observations over a prolonged period are required to assess this possibility. The mating of heterozygous *CCRs(10)^#4a^* mice resulted in homozygous, heterozygous, and WT mice born with the expected Mendelian inheritance (Fig. 2i).

### Evaluation of genome-wide target specificity in the *Recql5* mutant

**Fig. 3.**
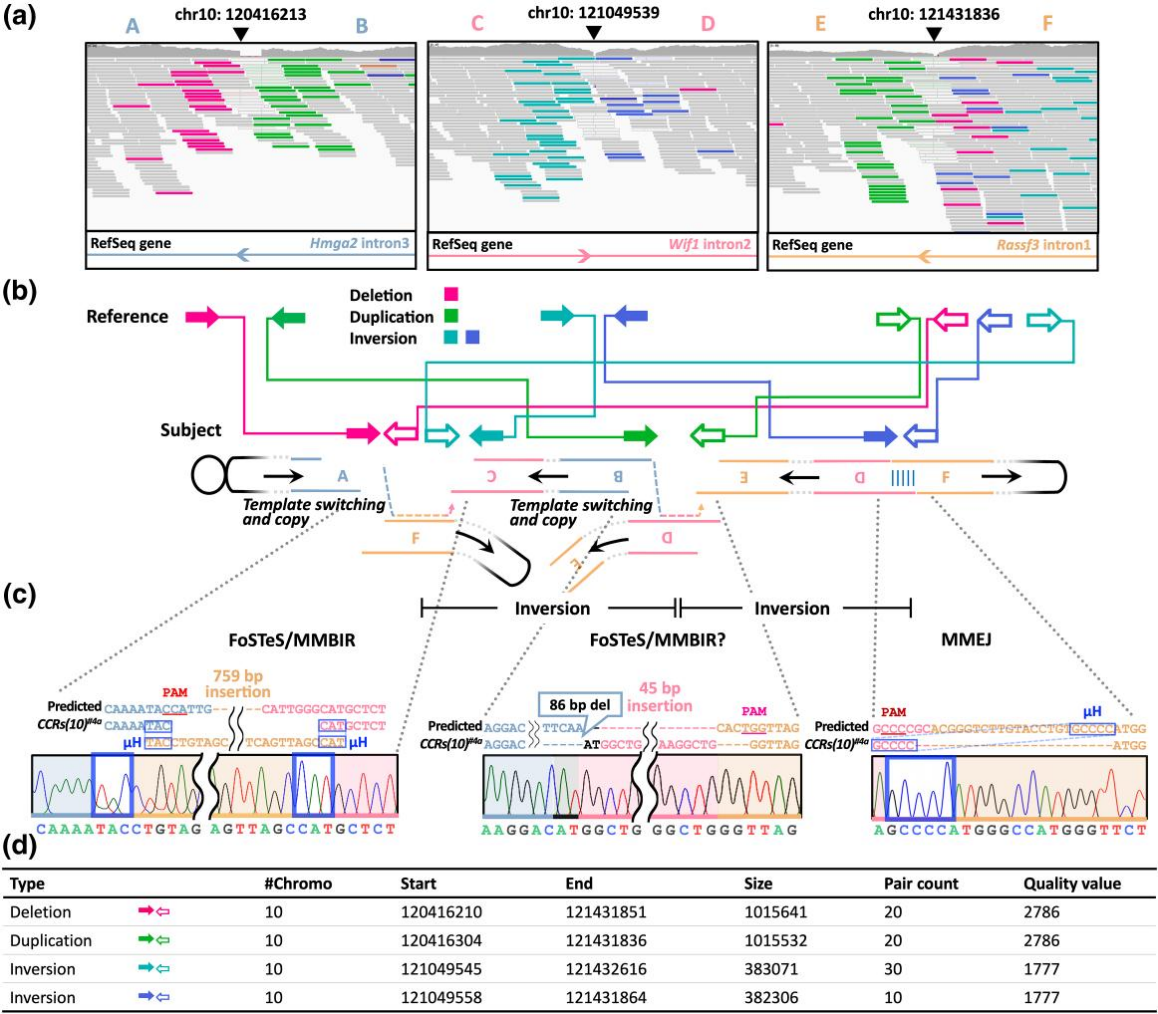
Validation of genome-wide target specificities in the *Recql5* mutant. a) WGS results. IGV browser of CCR data aligned to the mouse genome (mm10). The gRNA cut sites are shown using arrowheads. b) Schematic representation of paired-end read interpretation in IGV for complex rearrangements. Each arrow indicates the type and orientation of read pairs as aligned to the reference genome. c) Alignment of sequences from PCR products corresponding to the *Hmga2–Wif1*, *Hmga2–Rassf3*, and *Wif1–Rassf3* genomic breakpoint junctions. PAM, protospacer adjacent motif; μH, microhomology. d) Manta calls supporting chromosomal breakpoints.

We characterized the genomic structure of the mouse strains in detail using WGS. The *CCRs(10)^#4a^* strain showed multiple breakpoint junctions and was classified as deletion (red), duplication (green), and inversion (teal and blue) based on paired-ends with read depth changes (Fig. 3a and b). Sequencing of the breakpoint revealed microhomology patterns and sister chromatid-containing templates, wherein the added insertion was dependent on the Cas9 target site (Fig. 3c). Importantly, we demonstrated the induction of FoSTeS and MMBIR mechanisms in the *Recql5^em1/em1^* strain, replicating the 7.67-Mb inversion findings in the previous experiments (Fig. 1d). In addition to FoSTeS/MMBIR and tandem inversions, a 601-kb deletion was identified in the C*CRs(10)^#1^* strain (Supplementary Fig. 3a–c). Consistent with this result, the C*CRs(10)^#1^* strain did not express the fusion transcript corresponding to the middle of *Hmga2–Rassf3* (Fig. 2b), and no homozygotes were found (Supplementary Fig. 3e and f). The *CCRs(10)^#4b^* genome contained a 1,016-kb duplication harboring *Wif1* and *Rassf3* (Supplementary Fig. 4a–c). No discernible differences in the phenotype were observed between the *CCRs(10)^#4b/#4b^* and WT strains (Supplementary Fig. 4e and f).

However, HDR appeared to repair 2 of the 5 breakpoints examined in the WT background, while the others were likely repaired by microhomology-mediated end joining or nonhomologous end joining; however, no FoSTeS/MMBIR was observed (Supplementary Table 3). A complex genome architecture confuses the DNA repair machinery and induces template-switching events driven by FoSTeS and MMBIR (Liu et al. 2011; Pellestor and Gatinois 2018). Thus, loss of *Recql5* function may promote DNA repair machinery confusion and dictate the choice between the FoSTeS/MMBIR pathways.

These candidate rearrangement breakpoints were identified from WGS data using the Manta structural variation detection algorithm (Chen et al. 2016b); however, no target-independent complex rearrangements were identified by comparing the test genomes (Supplementary Figs. 3d and 4d). These results establish the efficiency and specificity of chromosomal engineering using the proposed approach.

### *Recql5* mutant mediates a broad pattern of chromosomal rearrangements

We generated CCRs on chromosome 2 that included topologically associating domains at the *HoxD* loci using the *i*-GONAD technique to test the broad applicability of the *Recql5* mutant approach. These loci are necessary to develop the proximal part of the limb, including the future arm and forearm (Acemel et al. 2016). We engineered CCRs comprising sequential inversions between *Atf2* neighborhood (*Atf2N*) and *Hoxd1N* and between *Hoxd1N* and *Nfe2l2N* to study the effects of the structural variants (Supplementary Fig. 5a). We screened F0 mice in the *Recql5^em1/em1^* strains using PCR amplification and DNA sequencing of 3 junction points and detected chromosomal rearrangements in 2 of the 4 F0 mice (Supplementary Fig. 5b and f). In one of the possible CCR mouse lines (named F0-#2), 2 HDR-repaired junction points were detected, whereas the other junction point was repaired with structural changes that could not be amplified by PCR (Supplementary Fig. 5b). In contrast, the control C57BL/6N strain did not show any breakpoints in the 7 pups (Supplementary Fig. 5f). Although the RNP-based CRISPR/Cas system is characterized by high-efficiency germline transmission (Chen et al. 2016a), F0-#2 did not transmit the targeted CCRs to their 34 offspring. In additional studies, we examined DNA extracted from the testes and semen of F0-#2 animals; however, the semen samples did not exhibit a PCR signal between *Atf2N* and *Nfe2l2N* (Supplementary Fig. 5c). In our experience, F0-#2 was the first mouse line wherein the mutation was not transmitted to the gametes using an RNP-based CRISPR/Cas system. Although we cannot accurately explain this transmission disturbance, asymmetric disjunction may result in a wide range of highly imbalanced gametes, many of which do not survive until the end of spermatogenesis (Li et al. 2013). Perhaps, breakpoints in intergenic regions may disturb the interactions between the promoter and transcriptional units with its *cis*-acting regulators through positional effects, thereby severely affecting gene expression (Liang et al. 2022).

In vitro electroporation and examination of individual zygotes showed that the *Recql5^em1/em1^* strain exhibited increased chromosomal rearrangement efficiency compared to the WT control (Supplementary Fig. 5g and Table 4). We selected the *Gprin3N*, *SncaN*, and *Grid2N* regions for the 3rd model of CCRs because changes in these areas are unlikely to be lethal, allowing us to study them without the risk of lethality (Supplementary Fig. 5d). The *Recql5^em1/em1^* strain also showed increased chromosomal rearrangement efficiency compared to the WT control (Supplementary Fig. 5g), although chromosomal breakpoints were exclusively detected at the left breakpoint for the *Gprin3N*, *SncaN*, and *Grid2N* target regions on chromosome 6 (Supplementary Fig. 5e and g). The distance between these target sites on the genome appears to influence the tendency for rearrangement, indicating that target gene loci that are farther apart are less likely to undergo inversion following DSBs induced by CRISPR/Cas9. A significant proportion of *Recql5^em1/em1^* strain embryos reached the blastocyst stage even after the induction of multiple DSBs (Supplementary Fig. 5g); this trend was also observed in the above experiments (Fig. 2d).

### Statistical significance of genetic rearrangements in the *Recql5* mutant

**Fig. 4.**
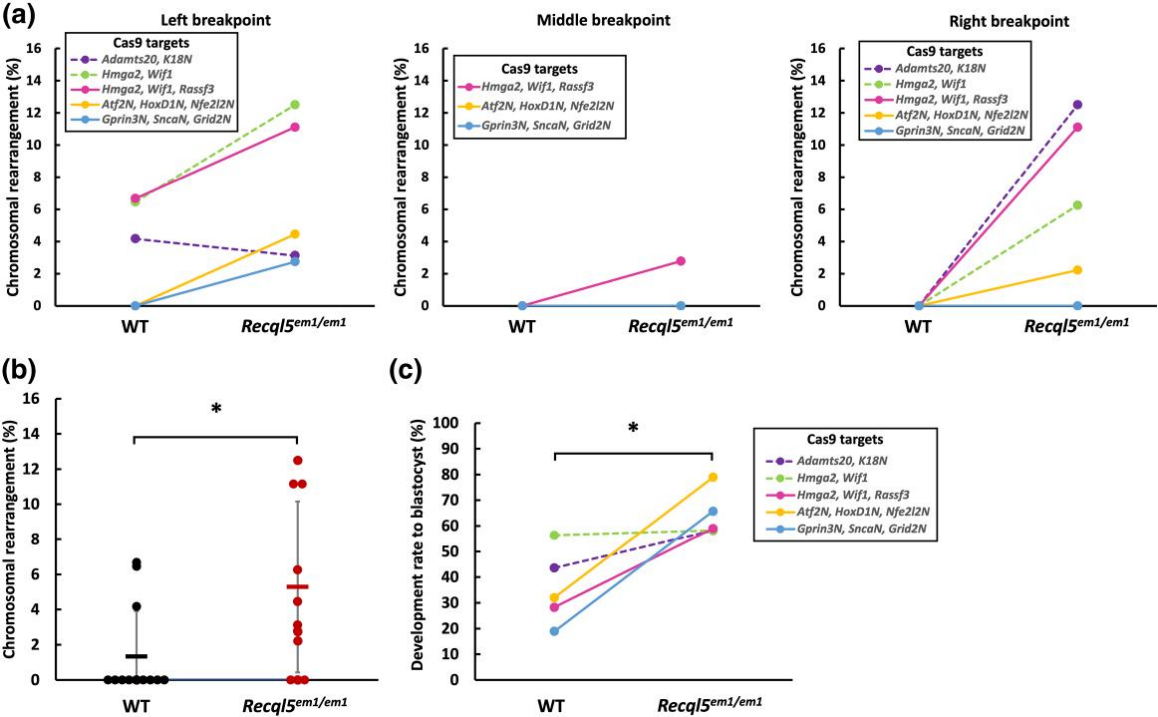
Efficiency of chromosomal rearrangement and blastocyst development rates following in vitro electroporation in the *Recql5* mutant. a) Chromosomal rearrangement efficiencies at 3 distinct breakpoints following in vitro electroporation in WT and *Recql5^em1/em1^* strains. The dashed lines indicate inversions, and the solid lines represent CCRs. b) Chromosomal rearrangement efficiency across all targeted gene loci. Results are presented as the mean ± SD, including data represented by scatter blots. *P < 0.05, Mann–Whitney *U* test (*n* = 13). c) Blastocyst development rate across all targeted gene loci. *P < 0.05, Mann–Whitney *U* test (*n* = 5). The dashed lines indicate inversions, and the solid lines represent CCRs.

In our comprehensive analysis of CRISPR/Cas9-mediated chromosomal rearrangements in the *Recql5^em1/em1^* strain, we evaluated the efficiency of targeted genetic modifications at various genomic loci (Fig. 4a). Our study demonstrated a notable enhancement in the overall efficiency of genetic modification in the *Recql5^em1/em1^* strain compared to the WT control group (*P* = 0.0158) through the application of the Mann–Whitney *U* test (Fig. 4b; Supplementary Table 5). Additionally, employing the same statistical method to evaluate the rate of development to the blastocyst stage revealed a statistically significant increase in the number of *Recql5^em1/em1^* strain embryos (*P* = 0.0079; Fig. 4c; Supplementary Table 5). The progression of a substantial number of *Recql5^em1/em1^* strain embryos to the blastocyst stage suggests the activation of alternative DNA repair mechanisms in these embryos that mitigate genomic instability and prevent cell death, despite the induction of multiple DSBs. For instance, several studies indicate that specific genetic mutations may promote error-prone backup pathways, potentially leading to changes in genomic structure and avoiding apoptosis (Roos et al. 2016; Groelly et al. 2023). The *Recql5^em1/em1^* mutant in tolerating genomic manipulation without compromising development to the blastocyst stage underscores its potential as a viable candidate for advanced genetic engineering applications. To the best of our knowledge, this is the first study to efficiently introduce CCRs into mouse zygotes using CRISPR-based methods. This approach opens new avenues for investigating the role of chromosomal rearrangements in diseases and provides a powerful tool for engineering genetic modifications in various organisms. The *Recql5^em1/em1^* strains generated in this study will be deposited at the RIKEN BioResource Research Center.

## Limitations of the study

Here, we describe a new genome editing method using a *Recql5* mutant mouse and show that it can efficiently induce various types of chromosomal rearrangements, including CCRs and inversions, in mouse zygotes. Although this technique has considerable potential, it is also associated with unexpected DSB repair mechanisms such as FoSTeS/MMBIR. Thus, the structural features of induced rearrangements are likely to depend on a variety of factors, such as features specific to the targeted genomic regions, the number of breakpoints, and the unique properties of mouse lines. In FoSTeS and MMBIR models, the absence of a homologous template during HDR possibly results in the activation of microhomology pairing repair of broken ends, a more error-prone DNA repair. Improvement of *Recql5* mutations may overcome these concerns. Specifically, the *Recql5* KIX mutation (Islam et al. 2010), which influences transcription stress regulation, along with the *Recql5*^Δ652–674^ and *Recql5*^F666A^ mutations (Schwendener et al. 2010), which do not disrupt RAD51 filaments, could enhance our CCR model mice. While a detailed exploration of these mutations exceeds the scope of this study, it is an essential direction for future research to improve genome editing precision and safety, especially for therapeutic uses.

## Supplementary Material

iyae054_Supplementary_Data

## Data Availability

The *Recql5^em1/em1^* mice used in this study are available at the RIKEN BioResource Research Center (RBRC12284). The WGS data reported in this paper were deposited into the DNA Data Bank of Japan Sequence Read Archive (https://ddbj.nig.ac.jp/DRASearch/, Accession: PRJDB15772). Supplemental material available at GENETICS online.
