# Supplementary material for: A Recql5 mutant facilitates complex CRISPR/Cas9-mediated chromosomal engineering in mouse zygotes
Source: Genetics. 2024 Apr 5;227(2):iyae054. doi: 10.1093/genetics/iyae054 (PMC11151919; doi:10.1093/genetics/iyae054)
Supplement: iyae054_Supplementary_Data [file iyae054_supplementary_data.pdf]

SUPPLEMENTARY INFORMATION

A *Recql5* mutant facilitates complex CRISPR/Cas9-mediated-chromosomal engineering in mouse zygotes

Satoru Iwata[1,2,3,4,#], Miki Nagahara[1], Risako Ido[2], Takashi Iwamoto [1,2]

[1]Center for Education in Laboratory Animal Research, Chubu University
[2]Department of Biomedical Sciences, College of Life and Health Sciences, Chubu University
[3]College of Bioscience and Biotechnology, Chubu University
[4]Center for Mathematical Science and Artificial Intelligence, Chubu University
[#]To whom correspondence should be addressed: satoru_iwata@isc.chubu.ac.jp

Supplementary Figures:
Supplementary Fig. 1: Equipment for *i*-GONAD of mouse zygotes.
Supplementary Fig. 2: Generation and phenotype of *Recql5* mutant mouse.
Supplementary Fig. 3: Validation of genome-wide target specificities in *CCRs(10)*$^{\#1}$ mice.
Supplementary Fig. 4: Validation of genome-wide target specificities in *CCRs(10)*$^{\#4b}$ mice.
Supplementary Fig. 5: Complex chromosomal engineering in mouse zygotes at the various genomic loci using the *Recql5* mutant.

Supplementary Tables:
Supplementary Table 1: Sequences of the *Adamts20–K18N* inversion genomic breakpoints obtained in WT and *Recql5.*
Supplementary Table 2: Sequences of the *Hmga2–Wif1* inversion genomic breakpoints obtained in WT and *Recql5* mutant mice.
Supplementary Table 3: Sequences of the *Hmga2–Wif1*, *Hmga2–Rassf3*, and *Wif1–Rassf3* genomic breakpoints obtained in WT and *Recql5* mutant mice.
Supplementary Table 4: Sequences of the *Atf2N–Hoxd1N*, *Atf2N–Nfe2l2N*, and *Hoxd1N–Nfe2l2N* genomic breakpoints obtained in *Recql5* mutant mice.
Supplementary Table 5: Chromosomal rearrangement and blastocyst development efficiencies following *in vitro* electroporation in WT and *Recql5* mutant zygotes.
Supplementary Table 6: gRNAs used in the present study.
Supplementary Table 7: Single-stranded oligodeoxynucleotides (ssODNs) used in the present study.
Supplementary Table 8: Primers used in the present study.

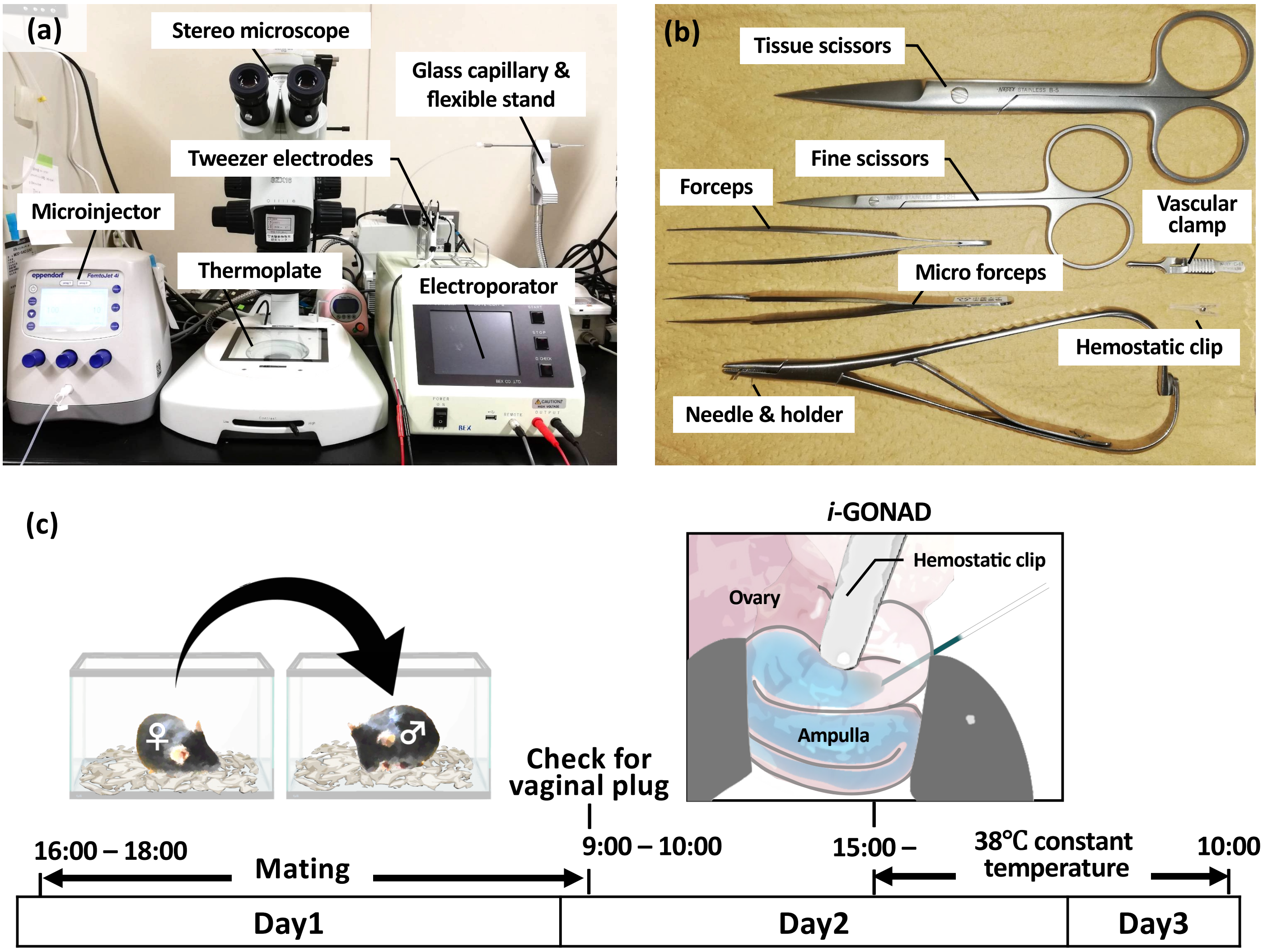

Supplementary Fig. 1: Equipment for *i*-GONAD of mouse zygotes.

a) The instruments used for both techniques—injection and electroporation. b) Surgical equipment. c) Experimental procedures for induction of CCRs using *i*-GONAD.

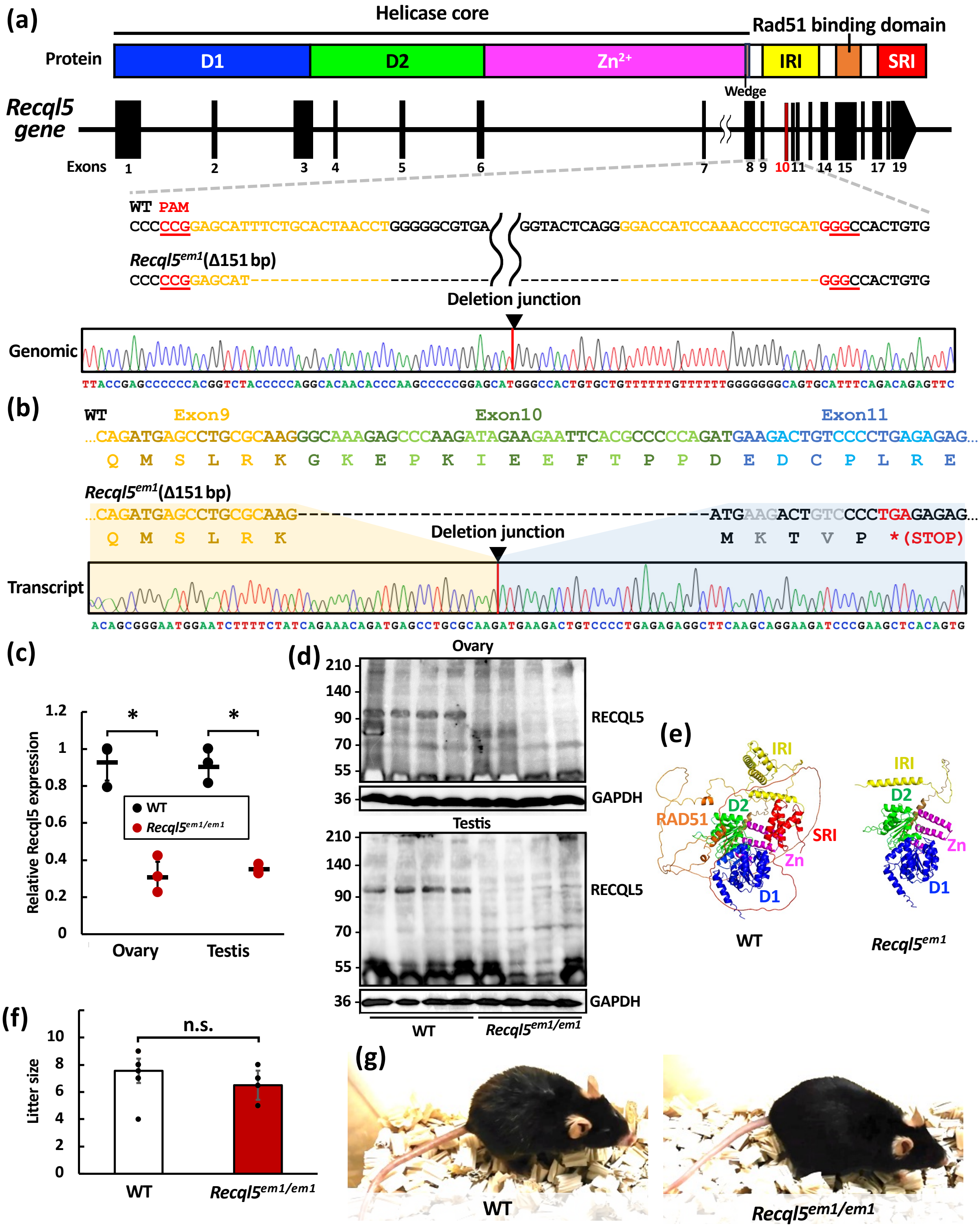

Supplementary Fig. 2: Generation and phenotype of *Recql5* mutant mouse.
a) Schematic representation of *Recql5* gene deletion and protein domain. The protospacer-adjacent motif (PAM) sequence is underlined in red. The gRNA sequence is indicated in yellow. b) Alignment of sequences corresponding to the *Recql5* cDNA. The *Recql5^{em1}* caused a frameshift mutation resulting in a premature stop codon. c) Quantitative RT-PCR analysis of *Recql5* expression in the ovaries and testes. Results are presented as means ± SD, including data represented by scatter blots. Error bars, mean ± S.D. *P < 0.05 (two-tailed Student's t-test) (n = 4). d) Western blot analysis for RECQL5 protein expression in the ovaries and testes. e) Predicted protein folding structures of WT and *Recql5^{em1}* protein using AlphaFold2, showing the disrupted domains owing to the mutation. f) Comparison of the litter sizes of WT (n = 9) and *Recql5$^{em1/em1}$* mice (n = 8). n.s., not significant. Error bars, mean ± S.D. g) Images of representative post-natal year 2 WT (left) and *Recql5$^{em1/em1}$* (right) male mice.

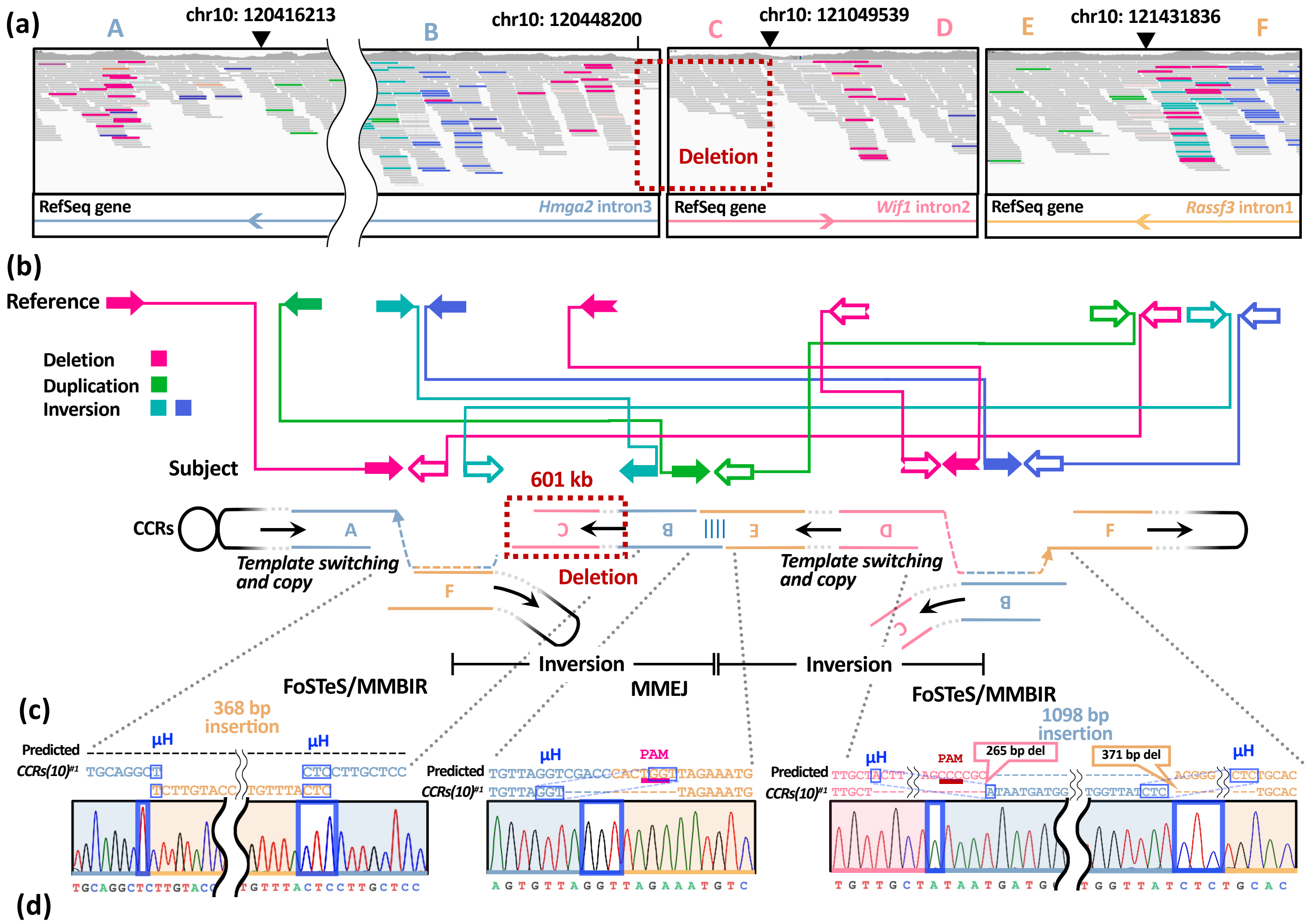

(d)

Type		#Chromo	Start	End	Size	Pair count	Quality value
Deletion	➡⇦	10	120415542	121431846	1016304	11	717
Deletion	➡⇦	10	120448107	121049828	601721	11	77
Duplication	➡⇦	10	120416224	121431833	1015609	4	717
Inversion	➡⇦	10	120447014	121432214	985200	11	1167
Inversion	➡⇦	10	120447015	121432215	985200	21	1167

(e)

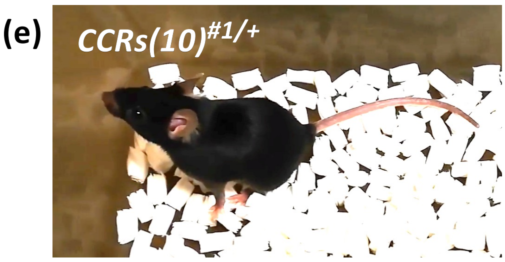

(f)

Genotype	Expected (*n*)	Observed (*n*)	Chi-square value	*P*-value
+/+	8.5	8	0.02941	
CCRs(10)$^{\#1/+}$	17	26	4.7647	
CCRs(10)$^{\#1/\#1}$	8.5	0	8.5	
**Total**	34	34	13.2941	0.001297

Supplementary Fig. 3: Validation of genome-wide target specificities in *CCRs(10)*$^{\#1}$ mice.

a) WGS results. IGV browser image of *CCRs(10)*$^{\#1}$ data aligned to the mouse genome (mm10). The gRNA cut sites are shown using arrowheads. b) Schematic representation of paired-end read interpretation in IGV for complex rearrangements. Each arrow indicates the type and orientation of read pairs as aligned to the reference genome. c) Alignment of sequences from PCR products corresponding to the *Hmga2–Wif1*, *Hmga2–Rassf3*, and *Wif1–Rassf3* genomic breakpoint junctions. PAM, protospacer adjacent motif; μH, microhomology. d) Manta calls supporting chromosomal breakpoints. e) Image of representative heterozygous *CCRs(10)*$^{\#1}$ mouse. f) Mendelian ratios of newborn mice from *CCRs(10)*$^{\#1}$ heterozygous crossings.

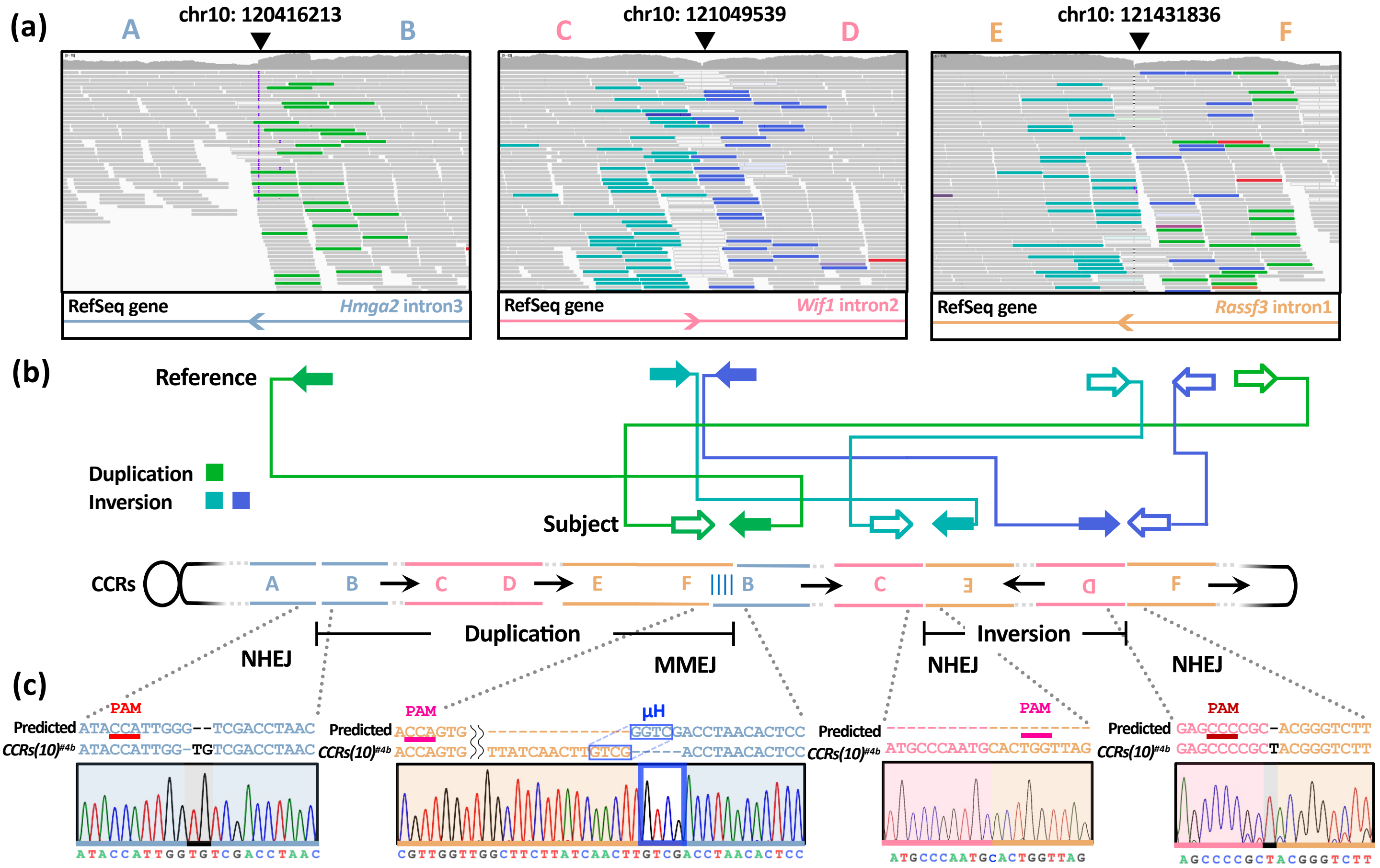

(d)

Type	#Chromo	Start	End	Size	Pair count	Quality value
Duplication	10	120416220	121432457	1016237	30	959
Inversion	10	121049493	121431816	382323	13	2635
Inversion	10	121049555	121431840	382285	58	1777

(e)

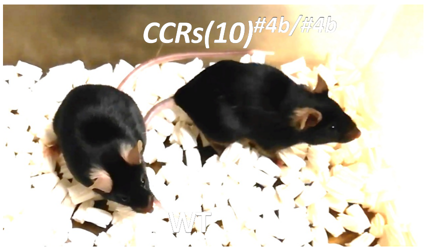

(f)

Genotype	Expected (*n*)	Observed (*n*)	Chi-square value	*P*-value
+/+	5	6	0.2	
CCRs(10)$^{\#4b/+}$	10	7	0.9	
CCRs(10)$^{\#4b/\#4b}$	5	7	0.8	
**Total**	20	20	1.9	0.38674102

Supplementary Fig. 4: Validation of genome-wide target specificities in *CCRs(10)*$^{\#4b}$ mice.

a) WGS results. IGV browser image of *CCRs(10)*$^{\#4b}$ data aligned to the mouse genome (mm10). The gRNA cut sites are shown using arrowheads. b) Schematic representation of paired-end read interpretation in IGV for complex rearrangements. Each arrow indicates the type and orientation of read pairs as aligned to the reference genome. c) Alignment of sequences from PCR products corresponding to the *Hmga2–Wif1*, *Hmga2–Rassf3*, and *Wif1–Rassf3* genomic breakpoint junctions. PAM, protospacer adjacent motif; μH, microhomology. d) Manta calls supporting chromosomal breakpoints. e) Images of representative wild-type (WT) and homozygous *CCRs(10)*$^{\#4b}$ mice. f) Mendelian ratios of newborn mice from *CCRs(10)*$^{\#4b}$ heterozygous crossings.

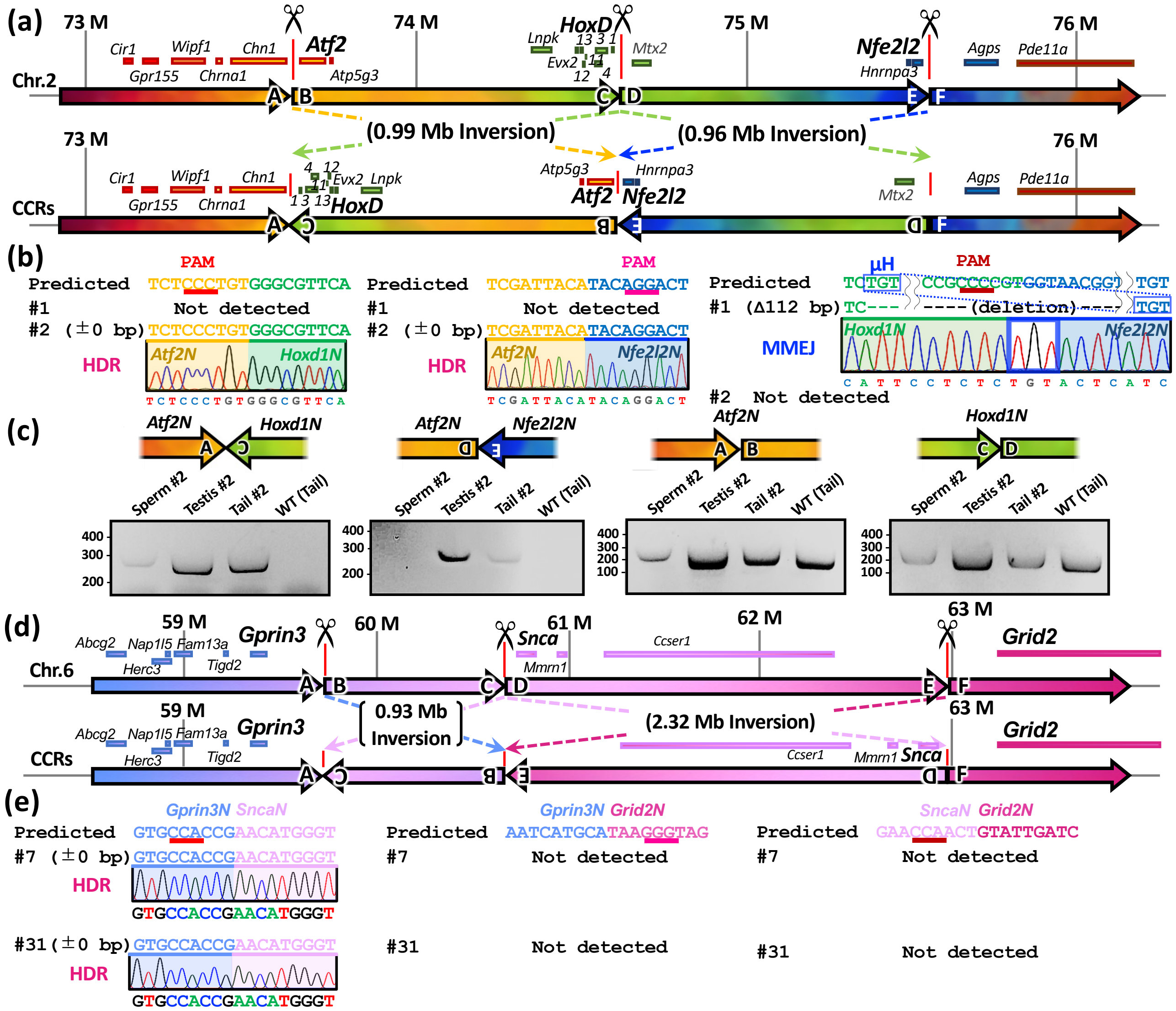

(f)

Cas9 targets	Distance (Mb)	Strain	Pregnant mice/ mice treated (*n*)	Pups born (*n*)	Chromosomal rearrangements (*n*): Left breakpoint detected	Middle breakpoint detected	Right breakpoint detected
Atf2N *HoxD1N* *Nfe2l2N*	1.95	WT	3/11	7	0	0	0
		Recql5$^{em1/em1}$	2/4	4	1	1	1

(g)

Cas9 targets	Distance (Mb)	Strain	Embryos treated (*n*)	No. of embryos develop into (*n*): 2-cell	4-cell	Blastocyst	Chromosomal rearrangements (*n*): Left breakpoint detected	Middle breakpoint detected	Right breakpoint detected
Atf2N *HoxD1N* *Nfe2l2N*	1.95	WT	53	25	17	17	0	0	0
		Recql5$^{em1/em1}$	57	49	45	45	2	0	1
Gprin3N *SncaN* *Grid2N*	3.25	WT	132	108	27	25	0	0	0
		Recql5$^{em1/em1}$	111	92	84	73	2	0	0

Supplementary Fig. 5: Complex chromosomal engineering in mouse zygotes at the various genomic loci using the *Recql5* mutant.

a) Schematic of the CCRs created between the *Atf2N*, *Hoxd1N*, and *Nfe2l2N* in chromosome 2. b) Alignment of the sequences corresponding to the *Atf2N–Hoxd1N*, *Atf2N–Nfe2l2N*, and *Hoxd1N–Nfe2l2N* genomic breakpoint junctions. c) PCR amplification of the breakpoint junctions in CCR mouse sperm, testes, tail, and WT. d) Schematic of the CCRs created between the *Gprin3N*, *SncaN*, and *Grid2N* in chromosome 6. e) Alignment of the sequences corresponding to the *Gprin3N–SncaN*, *Gprin3N–Grid2N*, and *SncaN–Grid2N* genomic breakpoint junctions. f) Summary of the experimental efficiency of CCRs using the *i*-GONAD technique. g) Summary of CCRs efficiency and embryonic development stages post *in vitro* electroporation.

Supplementary Table 1: Sequences of the *Adamts20–K18N* inversion genomic breakpoints obtained in WT and *Recql5.*

Experiment Type	strain	Left breakpoint<br>*Adamts20–K18N*	Right breakpoint<br>*Adamts20–K18N*
i-GONAD	WT	Predicted TGCTCTTGATTCCACCTCCGGGAGGGGATAATCAGTCAGGT<br>#8 (±0 bp) TGCTCTTGATTCCACCTCCGGGAGGGGATAATCAGTCAGGT<br>HDR 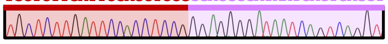<br>TGCTCTTGATTCCACCTCCGGGAGGGGATAATCAGTCAGGT	Not detected
in vitro EP	WT	Predicted TGCTCTTGATTCCACCTCCGGGAGGGGATAATCAGTCAGGT<br>#2 (±0 bp) TGCTCTTGATTCCACCTCCGGGAGGGGATAATCAGTCAGGT<br>HDR 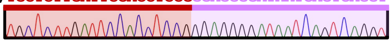<br>TGCTCTTGATTCCACCTCCGGGAGGGGATAATCAGTCAGGT	Not detected
in vitro EP	*Recql5$^{em1/em1}$*	Not detected	Predicted AGCTCGTGTTCAAGGACATGCGTAT ACGCATTTGATCCTC<br>#2 (Δ38 bp) AGCTCGTGTTCAAGGAC---------ACGCATTTGATCCTC<br>NHEJ 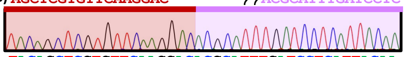<br>TACAGCTCGTGTTCAAGGACACGCATTTGATCCTCATTACAA
in vitro EP	*Recql5$^{em1/em1}$*	μH<br>Predicted CTCTTGATTCCACCTCCGGGAGGG TTCCTGCTTTGAGAAGAC<br>#12 (Δ133 bp) CTCTTGA--------------- TTCCTGCTTTGAGAAGAC<br>MMEJ 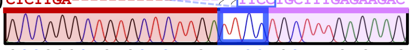<br>GCCGGGCTGTGCTCTTGATTCCTGCTTTGAGAAG	Not detected
in vitro EP	*Recql5$^{em1/em1}$*	Not detected	Predicted AGCTCGTGTTCAAGGACATGCGTAT ACGCATTTGATCCTC<br>#13 (Δ38 bp) AGCTCGTGTTCAAGGAC---------ACGCATTTGATCCTC<br>NHEJ 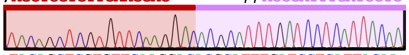<br>TACAGCTCGTGTTCAAGGACACGCATTTGATCCTCATTACAA
in vitro EP	*Recql5$^{em1/em1}$*	Not detected	Predicted AGCTCGTGTTCAAGGACATGCGTATACACAGGGCTTCGCAGT<br>#20 (±0 bp) AGCTCGTGTTCAAGGACATGCGTATACACAGGGCTTCGCAGT<br>HDR 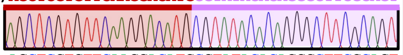<br>AGCTCGTGTTCAAGGACATGCGTATACACAGGGCTTCGCAGT

Supplementary Table 2: Sequences of the *Hmga2–Wif1* inversion genomic breakpoints obtained in WT and *Recql5* mutant mice.

Experiment Type	strain	Left breakpoint<br>*Hmga2–Wif1*	Right breakpoint<br>*Hmga2–Wif1*
i-GONAD	WT	Not detected	Predicted GAGTGTTAGGTCGACC----//----GCGGGGCTCTCAGTAA<br>#8 (Δ11+80 bp) GAGTGTTAGGTCG---GGCA//GATG---------CTCAGTAA<br>NHEJ<br>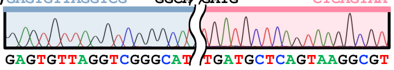<br>GAGTGTTAGGTCGGGCAT//TGATGCTCAGTAAGGCGT
in vitro EP	WT	Predicted GCACCCACCAAAATACCATTGCATTGGGCATGCTCTATTGAA<br>#5 (Δ12 bp) GCACCCACCAAAATACCAT------------GCTCTATTGAA<br>NHEJ<br>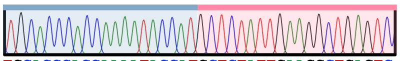<br>TGCACCCACCAAAATACCATGCTCTATTGAAGGCTGAGTC	Not detected
in vitro EP	WT	Predicted GCACCCACCAAAATACCATTGCATTGGGCATGCTCTATTGAA<br>#15 (±0 bp) GCACCCACCAAAATACCATTGCATTGGGCATGCTCTATTGAA<br>HDR<br>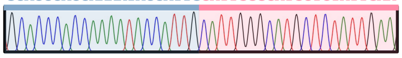<br>GCACCCACCAAAATACCATTGCATTGGGCATGCTCTATTGAA	Not detected
in vitro EP	*Recql5*$^{em1/em1}$	μH<br>Predicted GCACCCACCAAAATACCATTGCATTGGGCATGCTCTATTGAA<br>#5 (Δ5 bp) GCACCCACCAAAATACCATTG-----GGCATGCTCTATTGAA<br>MMEJ<br>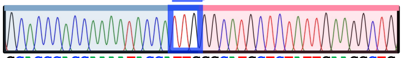<br>GCACCCACCAAAATACCATTGGGCATGCTCTATTGAAGGCTG	Not detected
in vitro EP	*Recql5*$^{em1/em1}$	μH<br>Predicted GCACCCACCAAAATACCATTGCATTGGGCATGCTCTATTGAA<br>#13 (Δ5 bp) GCACCCACCAAAATACCATTG-----GGCATGCTCTATTGAA<br>MMEJ<br>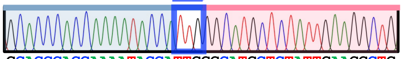<br>GCACCCACCAAAATACCATTGGGCATGCTCTATTGAAGGCTG	μH<br>Predicted AAAGGGAGTGTTAGGTCGACCGCGGGGCTCTCAGTAAGGCGT<br>#13 (Δ11 bp) AAAGGGAGTGTTAGG-----------GCTCTCAGTAAGGCGT<br>MMEJ<br>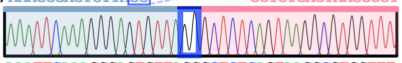<br>AAATTCAAAGGGAGTGTTAGGGCTCTCAGTAAGGCGTGGTTT
in vitro EP	*Recql5*$^{em1/em1}$	Predicted GCACCCACCAAAATACCATTGCATTGGGCATGCTCTATTGAA<br>#14 (Δ17 bp) GCACCCACCAAAATAC-----------------TCTATTGAA<br>NHEJ<br>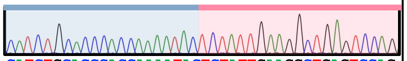<br>CATCTGCACCCACCAAAATACTCTATTGAAGGCTGAGTCCAG	Not detected
in vitro EP	*Recql5*$^{em1/em1}$	μH<br>Predicted GCACCCACCAAAATACCATTGCATTGGGCATGCTCTATTGAA<br>#18 (Δ12 bp) GCACCCACCAAAATACCAT------------GCTCTATTGAA<br>MMEJ<br>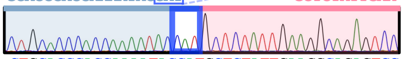<br>CTGCACCCACCAAAATACCATGCTCTATTGAAGGCAGAGTCC	Not detected
in vitro EP	*Recql5*$^{em1/em1}$	Not detected	Predicted AAAGGGAGTGTTAGGTCGACCGCGGGGCTCTCAGTAAGGCGT<br>#25 (Δ8 bp) AAAGGGAGTGTTA--------GCGGGGCTCTCAGTAAGGCGT<br>NHEJ<br>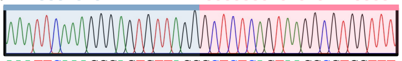<br>AAATTCAAAGGGAGTGTTAGGGCTCTCAGTAAGGCGTGGTTT

Supplementary Table 3: Sequences of the *Hmga2–Wif1*, *Hmga2–Rassf3*, and *Wif1–Rassf3* genomic breakpoints obtained in WT and *Recql5* mutant mice.

Experiment Type	Strain	Left breakpoint<br>*Hmga2–Wif1*	Middle breakpoint<br>*Hmga2–Rassf3*	Right breakpoint<br>*Wif1–Rassf3*
i-GONAD	WT	Not detected	μH<br>Predicted TGTTAGGTCGACCCACTGGTTAGAAATG<br>#2(Δ12 bp) TGTTAGGT------------TAGAAATG<br>MMEJ 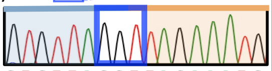<br>G T G T T A G G T T A G A A A T G	Predicted CTGAGAGCCCCGCACGGGTCTTGTAC<br>#2 (±0 bp) CTGAGAGCCCCGCACGGGTCTTGTAC<br>HDR 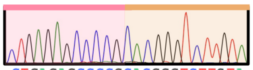<br>CTGAGAGCCCCGCACGGGTCTTGTA
i-GONAD	WT	Not detected	Not detected	Predicted CTGAGAGCCCCGCACGGGTCTTGTAC<br>#5 (±0 bp) CTGAGAGCCCCGCACGGGTCTTGTAC<br>HDR 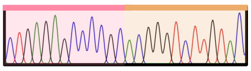<br>CTGAGAGCCCCGCACGGGTCTTGTA
i-GONAD	WT	Predicted AAATACCATTGCATTGGG ⟩⟩ TGGGCATGCT<br>#9(Δ44 bp) AAATACCATT--------- ⟩⟩ TGGGCATGCT<br>NHEJ 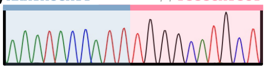<br>A A A T A C C A T T T G G G C A T G C T	Not detected	Predicted GAGCCCCGC------ACGGGTCTTGTA<br>#9(Δ9+5 bp) GAGCCCCG-ATTTT----GTCTTGTA<br>NHEJ 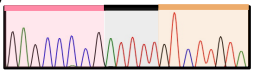<br>G A G C C C C G A T T T T G T C T T G T A
in vitro EP	WT	Predicted AATACCATTG---//---CATTGGGCAT<br>#5 (+40 bp) AATACCATTGCAT//CTCCATTGGGCAT<br>NHEJ 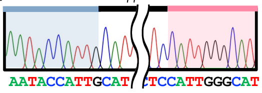<br>AATACCATTGCAT // CTCCATTGGGCAT	Not detected	Not detected
in vitro EP	*Recql5*$^{em1/em1}$	Predicted CAAAATACCATTGCATTGGGCATGCT<br>#12 (Δ6 bp) CAAAATACCATTG------GCATGCT<br>NHEJ 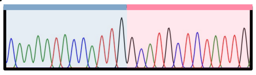<br>CAAAATACCATTGGCATGCTCTATTG	μH<br>Predicted GTTAGGTCGACCCACTGGTTAGAAAT<br>#12 (Δ4 bp) GTTAGGTCGAC----TGGTTAGAAAT<br>MMEJ 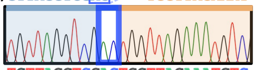<br>TGTTAGGTCGACTGGTTAGAAATGTC	Not detected
in vitro EP	*Recql5*$^{em1/em1}$	Not detected	Not detected	Predicted ACTGAGAGCCCCGCACGGGTCTTGTAC<br>#13 (±0 bp) ACTGAGAGCCCCGCACGGGTCTTGTAC<br>HDR 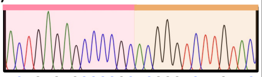<br>ACTGAGAGCCCCGCACGGGTCTTGTAC
in vitro EP	*Recql5*$^{em1/em1}$	Not detected	Not detected	Predicted ACTGAGAGCCCCGCACGGGTCTTGTAC<br>#14 (±0 bp) ACTGAGAGCCCCGCACGGGTCTTGTAC<br>HDR 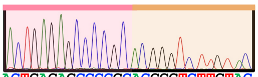<br>ACTGAGAGCCCCGCACGGGTCTTGTAC
in vitro EP	*Recql5*$^{em1/em1}$	Predicted CAAAATACCATTGCATTGGGCATGCT<br>#15 (±0 bp) CAAAATACCATTGCATTGGGCATGCT<br>HDR 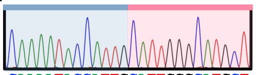<br>CAAAATACCATTGCATTGGGCATGCT	Not detected	Not detected
in vitro EP	*Recql5*$^{em1/em1}$	μH<br>Predicted CAAAATACCATTGCATTGGGCATGCT<br>#17 (Δ5 bp) CAAAATACCATTG-----GGCATGCT<br>MMEJ 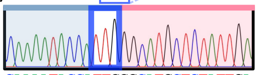<br>CAAAATACCATTGGGCATGCTCTATTGA	Not detected	Not detected
in vitro EP	*Recql5*$^{em1/em1}$	Predicted CAAAATACCATTGCATTGGGCATGCT<br>#20 (±0 bp) CAAAATACCATTGCATTGGGCATGCT<br>HDR 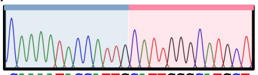<br>CAAAATACCATTGCATTGGGCATGCT	Not detected	Predicted ACTGAGAGCCCCGCACGGGTCTTGTAC<br>#20 (±0 bp) ACTGAGAGCCCCGCACGGGTCTTGTAC<br>HDR 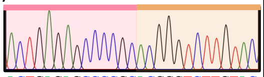<br>ACTGAGAGCCCCGCACGGGTCTTGTAC
in vitro EP	*Recql5*$^{em1/em1}$	Not detected	Not detected	Predicted ACTGAGAGCCCCGCACGGGTCTTGTAC<br>#29 (±0 bp) ACTGAGAGCCCCGCACGGGTCTTGTAC<br>HDR 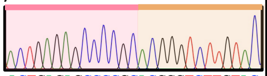<br>ACTGAGAGCCCCGCACGGGTCTTGTAC

Supplementary Table 4: Sequences of the *Atf2N–Hoxd1N*, *Atf2N–Nfe2l2N*, and *Hoxd1N–Nfe2l2N* genomic breakpoints obtained in *Recql5* mutant mice.

Experiment Type	Strain	Left breakpoint *Atf2N–Hoxd1N*	Middle breakpoint *Atf2N–Nfe2l2N*	Right breakpoint *Hoxd1N–Nfe2l2N*
in vitro EP	*Recql5*$^{em1/em1}$	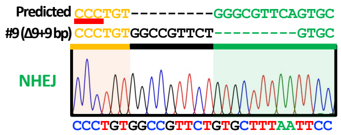	**Not detected**	**Not detected**
in vitro EP	*Recql5*$^{em1/em1}$	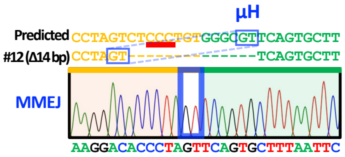	**Not detected**	**Not detected**
in vitro EP	*Recql5*$^{em1/em1}$	**Not detected**	**Not detected**	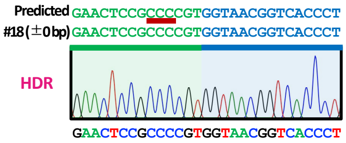

Supplementary Table 5: Chromosomal rearrangement and blastocyst development efficiencies following *in vitro* electroporation in WT and *Recql5* mutant zygotes.

Cas9 targets	Distance (Mb)	Strain	Embryos treated (*n*)	Blastocyst Development Rate (%) (Number of Blastocysts/Total Embryos)	Percentage of Chromosomal Rearrangements (%) (Number with Detected Rearrangement/Total Blastocysts)		
					Left breakpoint detected	Middle breakpoint detected	Right breakpoint detected
Adamts20<br>*K18N*	7.67	WT	55	24/55 (43.6)	1/24 (4.17)		0/24 (0)
		$Recql5^{em1/em1}$	55	32/55 (58.2)	1/32 (3.12)		4/32 (12.5)
Hmga2<br>*Wif1*	0.63	WT	55	31/55 (56.4)	2/31 (6.45)		0/31 (0)
		$Recql5^{em1/em1}$	55	32/55 (58.2)	4/32 (12.5)		2/32 (6.25)
Hmga2<br>*Wif1*<br>*Rassf3*	1.01	WT	53	15/53 (28.3)	1/15 (6.67)	0/15 (0)	0/15 (0)
		$Recql5^{em1/em1}$	61	36/61 (59.0)	4/36 (11.1)	1/36 (2.78)	4/36 (11.1)
Atf2N<br>*HoxD1N*<br>*Nfe2l2N*	1.95	WT	53	17/53 (32.1)	0/17 (0)	0/17 (0)	0/17 (0)
		$Recql5^{em1/em1}$	57	45/57 (78.9)	2/45 (4.44)	0/45 (0)	1/45 (2.22)
Gprin3N<br>*SncaN*<br>*Grid2N*	3.25	WT	132	25/132(18.9)	0/25 (0)	0/25 (0)	0/25 (0)
		$Recql5^{em1/em1}$	111	73/111(65.8)	2/73 (2.74)	0/73 (0)	0/73 (0)

Supplementary Table 6: gRNAs used in the present study.

Target loci	Genomic location (mm10/GRCm38)	Target Sequences (PAM)	CHOPCHOP (https://chopchop.cbu.uib.no/) Number of mismatches: 0	1	2	3	Efficiency
Recql5	chr11:115896078	AGGTTAGTGCAGAAATGCTC (CGG)	0	0	2	6	46.87
Recql5	chr11:115896218	GGACCATCCAAACCCTGCAT (GGG)	0	0	0	2	57.44
Adamts20	chr15:94347708	TCGTGTTCAAGGACATGCGG (AGG)	0	0	1	3	71.44
K18N	chr15:102034830	GAAGCCCTGTGTATACGGGA (GGG)	0	0	1	4	61.49
Hmga2	chr10:120416213	GGAGTGTTAGGTCGACCCAA (TGG)	0	0	0	0	67.29
Wif1	chr10:121049539	ATAGAGCATGCCCAATGGCG (GGG)	0	0	0	0	60.84
Rassf3	chr10:121431836	ACAGGTACAAGACCCGTCAC (TGG)	0	0	0	2	59.01
Atf2N	chr2:73802063	AGCAAGGGTCGATTACAACA (GGG)	0	0	0	0	70.21
HoxD1N	chr2:74789654	TAAAGCACTGAACGCCCACG (GGG)	0	0	0	0	72.43
Nfe2l2N	chr2:75750986	TCAGGGTGACCGTTACCTAC (AGG)	0	0	0	0	57.25
Gprin3N	chr6:59752018	CACTAAGTAATCATGCACGG (TGG)	0	0	0	0	76.29
SncaN	chr6:60662244	ACTATCGAACCCATGTTAGT (TGG)	0	0	0	1	51.24
Grid2N	chr6:62986069	TACTGAGCGATCAATACTAA (GGG)	0	0	0	0	54.99

Supplementary Table 7: Single-stranded oligodeoxynucleotides (ssODNs) used in the present study.

Target loci	Target Sequences (5′–3′)
Adamts20–*K18N* (Left)	CGTACTCGGGCCGGTCACAGAGCCGGGCTGTGCTCTTGATTCCACCTCCGGGAGGGGATAATCAGTCAGGTGCCTCGGCTCAGGTTTCTCGGGTCAGCTT
Adamts20–*K18N* (Right)	GATGGCGAGTGGGGACCATGGGGACCCTACAGCTCGTGTTCAAGGACATGCGTATACACAGGGCTTCGCAGTTCCCAGGGCTCTTACGCATTTGATCCTC
Hmga2–*Wif1* (Left)	CGTTGCAGCATGTAGGTGTGGTAGCATCTGCACCCACCAAAATACCATTGCATTGGGCATGCTCTATTGAAGGCTGAGTCCAGAGCTCTCCATTGGGCAT
Hmga2–*Wif1* (Right)	GAAGCACGAAGAAAAGAGTTTAAAAATTCAAAGGGAGTGTTAGGTCGACCGCGGGGCTCTCAGTAAGGCGTGGTTTTTTAAAAAAATCTTTTTTGGGTTA
Hmga2–*Wif1* (Left)	C*G*TTGCAGCATGTAGGTGTGGTAGCATCTGCACCCACCAAAATACCATTGCATTGGGCATGCTCTATTGAAGGCTGAGTCCAGAGCTCTCCATTGGGC*A*T
Hmga2–*Rassf3* (Middle)	G*A*AGCACGAAGAAAAGAGTTTAAAAATTCAAAGGGAGTGTTAGGTCGACCCACTGGTTAGAAATGTCGGGATTGGTTGCCCCCTGCCTCCTAGGCACT*G*G
Wif1–*Rassf3* (Right)	T*A*ACCCAAAAAAGATTTTTTTAAAAAACCACGCCTTACTGAGAGCCCCGCACGGGTCTTGTACCTGTAGCCCCATGGGCCATGGGTTCTTGGCTAATT*T*T
Atf2N–*Hoxd1N* (Left)	C*A*GCTCAAACAACATAATGGCAGCAGGGAAGGACACCCTAGTCTCCCTGTGGGCGTTCAGTGCTTTAATTCCGTGTTTGTCACAGCGATTGCTTGACT*C*T
Atf2N–*Nfe2l2N* (Middle)	A*G*GCTACAAGACAAAAAGACCACAGGGAATTCTAGCAAGGGTCGATTACATACAGGACTGTGGGACTAGTGAGTGGGATTCGGGCGCAGAAGTAGCTA*T*G
Hoxd1N–*Nfe2l2N* (Right)	T*T*ATTATTCATTCCTCTCTGTACTTCACCTGATATTGAACTCCGCCCCGTGGTAACGGTCACCCTGACTTTTAATGTCATAGATTGATTTCATCAGAT*G*T
Gprin3N–*SncaN* (Left)	C*T*GCCCGCCTCTGCCTCCGGAGTGCTTTGGATAAAAGGTGTGTGCCACCGAACATGGGTTCGATAGTGTCTATAGAGGCTGAACCACCAACCAAAGAG*T*A
Gprin3N–*Grid2N* (Middle)	A*C*GTATATTTACCTTGCTGGGACAATTATAAAGCACTAAGTAATCATGCATAAGGGTAGCACAGCCTGAGGCTGAAAAAATAGTTATTGGACAGAAAT*C*C
SncaN–*Grid2N* (Right)	A*G*GGAGTTGGAGGCGTCAAGGCCAGCACAAGAAGACCTACAGAACCAACTGTATTGATCGCTCAGTAGTCTTGCAATTAATCTTCTTGGCCAAGTATG*G*T

Supplementary Table 8: Primers used in the present study.

Target loci	Genomic location (mm10/GRCm38)	Sequences (5'–3')
**Genotyping primers**		
Recql5-F	chr11: 115895976 to 115895997	TCTTCATCTGAGAAAAGGGAGG
Recql5-R	chr11: 115896306 to 115896327	CTGGAGTACAAGGCCAGAGACT
Adamts20-1st-F	chr15: 94347286 to 94347307	AGGAGGGACATCAGGTTACAGA
Adamts20-1st-R	chr15: 94348147 to 94348168	TGTGATGACCACTGCATTATGA
Adamts20-2nd-F	chr15: 94347562 to 94347583	TCTCTGAAACTCGCAGACTGAC
Adamts20-2nd-R	chr15: 94347823 to 94347844	TTCCTGTGTGTGTGCTTCTTCT
K18N-1st-F	chr15: 102034486 to 102034507	CCTTTGTTCCGTTGATCATGTA
K18N-1st-R	chr15: 102035201 to 102035222	GAAACTGGAGGGTCACAGGTAG
K18N-2nd-F	chr15: 102034680 to 102034700	GGCATCAAATGTGTCTTCTCA
K18N-2nd-R	chr15: 102034945 to 102034966	TGATGTCTGTGGCCTTTACTGT
Hmga2-1st-F	chr10: 120415727 to 120415748	CCCCAGGGAAGGTAAATAATGT
Hmga2-1st-R	chr10: 120416637 to 120416658	TTGCTCTGGACAACATTCATTC
Hmga2-2nd-F	chr10: 120416078 to 120416099	GTCTGCCATGATGTTTGTTAGC
Hmga2-2nd-R	chr10: 120416271 to 120416292	AAGTGTTGAAGAGCAGAAAGGC
Wif1-1st-F	chr10: 121049111 to 121049132	TGACCCTCCACCATTAAAATTC
Wif1-1st-R	chr10: 121049971 to 121049992	AGATCATTGCTGGGAAGAAGAG
Wif1-2nd-F	chr10: 121049423 to 121049444	GTTCAGTAGCTGGAGGAGGATG
Wif1-2nd-R	chr10: 121049610 to 121049631	GGCTTTTTATGAAAGGCAACTG
Rassf3-1st-F	chr10: 121431397 to 121431418	GAAGACAGAGACAAACCATGCC
Rassf3-1st-R	chr10: 121432300 to 121432321	CTCTTTGTGGCACTTCTGTGTC
Rassf3-2nd-F	chr10: 121431721 to 121431742	GAGATTGCTCAGGACAGAGTGA
Rassf3-2nd-R	chr10: 121431917 to 121431944	TGTACTCATACAAAATTAATCACAAGGG
Atf2N-1st-F	chr2: 73801647 to 73801668	GACTAGAAGGTGGGACATCCTG
Atf2N-1st-R	chr2: 73802452 to 73802473	CCTGTTTCCTGATACAAGAGGC
Atf2N-2nd-F	chr2: 73802000 to 73802021	CTCTTCGCAAGACACATTTCAG
Atf2N-2nd-R	chr2: 73802244 to 73802265	CAGGCGTATTGGAAGACTTAGG
HoxD1N-1st-F	chr2: 74789228 to 74789249	CTGTTAGGGTTGTGAGGGAAAG
HoxD1N-1st-R	chr2: 74790112 to 74790133	AGCCTTTGATGCTAGCTGTCTC
HoxD1N-2nd-F	chr2: 74789496 to 74789517	GAGTGAACTGCAAGGAGGTTTC
HoxD1N-2nd-R	chr2: 74789720 to 74789741	CCAGGAACTTCTCTGCTTCTCT
Nfe2l2N-1st-F	chr2: 75750568 to 75750589	AGGTGGTGTAGACTGGTGGTCT
Nfe2l2N-1st-R	chr2: 75751453 to 75751474	GAGCAGTCAGTGCTTTTAAGGG
Nfe2l2N-2nd-F	chr2: 75750911 to 75750932	TTCCAAATCAAGAGTGGAACAA
Nfe2l2N-2nd-R	chr2: 75751125 to 75751146	AAACTAAACCACACCACAGCCT
Gprin3N-1st-F	chr6: 59751624 to 59751645	GGAGCGTAGGAGAGAACTTTTG
Gprin3N-1st-R	chr6: 59752410 to 59752431	TGCTCAAAACAATGATGAAACC
Gprin3N-2nd-F	chr6: 59751916 to 59751937	TAGCCCTGGATGTCCTGTAACT
Gprin3N-2nd-R	chr6: 59752155 to 59752176	TAGGGATCACAAATTGACATGG
SncaN-1st-F	chr6: 60661892 to 60661913	ACAGTCCCTCCTGTCTTCACTC
SncaN-1st-R	chr6: 60662618 to 60662638	CCTGTGACCAGTCAGGACTTC
SncaN-2nd-F	chr6: 60662006 to 60662027	GCACTGGCACATTAAGTCACAT
SncaN-2nd-R	chr6: 60662369 to 60662390	TATCTAGCAGCTGATGGAAGCA
Grid2N-1st-F	chr6: 62985641 to 62985662	GAAGCCTCTCTGTCCAGTGAAT
Grid2N-1st-R	chr6: 62986499 to 62986522	TCAAAATTCCAGTGATCTCCTACA
Grid2N-2nd-F	chr6: 62985938 to 62985959	ACACATATGCCTCAGAGCAAGA
Grid2N-2nd-R	chr6: 62986197 to 62986218	GAAAACCTTGGCTGGATCTAAA
**RT-PCR primers**		
RECQL5-Exon9-F	chr11: 115896851 to 115896872	TGATGAAGGTTCTGGAGGTAGC
RECQL5-Exon11-R	chr11: 115895792 to 115895813	TGGTTACTGATCAGAGCCTCCT
HMGA2-Exon4-F	chr10: 120374678 to 120374700	TTCTTCTGAACGACTTGTTGTGG
HMGA2-Exon2-R	chr10: 120473262 to 120473285	CAAAAACAAGAGCCCCTCTAAAGC
WIF1-Exon1-F	chr10: 121034441 to 121034462	GGAGAGCTTGTACCTGTGGATC
WIF1-Exon4-R	chr10: 121082144 to 121082164	CGTCTTGTTTGCCGAGACACG
RASSF3-Exon2-F	chr10: 121417140 to 121417161	TTGATTTCCTCTTTGCTGAGGT
RASSF3-Exon1-R	chr10: 121476034 to 121476054	GACCTCCTTCTTCAGGAGAGC
**qRT-PCR primers**		
RECQL5-Exon2-F	chr11: 115930733 to 115930754	TCTCTGTGCTATCAACTCCCTG
RECQL5-Exon3-R	chr11: 115896306 to 115896327	CCTGTACCGAGAGCTTTGAGTT
YWHAZ-Exon1-F	chr15: 36790806 to 36790828	CGTTGTAGGAGCCCGTAGGTCAT
YWHAZ-Exon2-R	chr15: 36776326 to 36776345	TCTGGTTGCGAAGCATTGGG
